# Recognition of MR1-antigen complexes by TCR Vγ9Vδ2

**DOI:** 10.3389/fimmu.2025.1519128

**Published:** 2025-02-18

**Authors:** José Pedro Loureiro, Alessandro Vacchini, Giuliano Berloffa, Jan Devan, Verena Schaefer, Vladimir Nosi, Rodrigo Colombo, Aisha Beshirova, Giulia Montanelli, Benedikt Meyer, Timothy Sharpe, Andrew Chancellor, Mike Recher, Lucia Mori, Gennaro De Libero

**Affiliations:** ^1^ Experimental Immunology, Department of Biomedicine, University Hospital and University of Basel, Basel, Switzerland; ^2^ Immunodeficiency Laboratory, Department of Biomedicine, University Hospital and University of Basel, Basel, Switzerland; ^3^ Biozentrum, University of Basel, Basel, Switzerland

**Keywords:** MR1, TCR γδ, Vγ9Vδ2, adaptive immunity, antigen recognition

## Abstract

The TCR-mediated activation of T cells expressing the TCR Vγ9Vδ2 relies on an innate-like mechanism involving the butyrophilin 3A1, 3A2 and 2A1 molecules and phospho-antigens, without the participation of classical antigen-presenting molecules. Whether TCR Vγ9Vδ2 cells also recognize complexes composed of antigens and antigen-presenting molecules in an adaptive-like manner is unknown. Here, we identify MR1-autoreactive cells expressing the TCR Vγ9Vδ2. This MR1-restricted response is antigen- and CDR3δ-dependent and butyrophilin-independent. TCR gene transfer reconstitutes MR1-antigen recognition, and engineered TCR Vγ9Vδ2 tetramers interact with soluble MR1-antigen complexes in an antigen-dependent manner. These cells are present in healthy individuals with low frequency and are mostly CD8^+^ or CD4-CD8 double negative. We also describe a patient with autoimmune symptoms and TCR γδ lymphocytosis in which ~10% of circulating T cells are MR1-self-reactive and express a TCR Vγ9Vδ2. These cells release pro-inflammatory cytokines, suggesting a possible participation in disease pathogenesis. Thus, MR1-self-antigen complexes can interact with some TCRs Vγ9Vδ2, promoting full cell activation and potentially contributing to diseases.

## Introduction

The MHC-related molecule 1 (MR1) is a non-classical MHC Class I-like molecule ubiquitously expressed and characterized by low polymorphism (1, 2). So far, MR1-restricted T cells have mainly been reported to express TCRs αβ and are grouped as i) Mucosal-associated invariant T (MAIT) cells, expressing semi-invariant TCRs (3–5) that recognize modified precursors of riboflavin, including ribityllumazines (6) and 5-(2-oxopropylideneamino)-6-D-ribitylaminouracil (5-OP-RU) (7); and ii) self-reactive MR1-restricted T (MR1T) cells (8–10), expressing polyclonal TCRs and activated by endogenous carbonyl adducts of nucleobases (11).

Rare TCR γδ cells were also shown to interact with MR1 tetramers loaded with 5-OP-RU or acetyl 6-formyl pterin (Ac-6-FP) (12). Most of these cells express the Vδ1 chain, and some the Vδ3 or Vδ5 chains. Among these MR1-tetramer-binding cells, these three chains paired mainly with the Vγ8 chain and much less frequently with Vγ2-5 and Vγ9 chains (12). In two studies, 5-OP-RU-MR1 tetramers were reported to bind <0.1% of TCR Vδ2-expressing cells from circulating blood (12, 13), but no functional data complemented this staining yet. The crystal structures of one Vδ1-MR1 (12) and one Vδ3-MR1 (14) binary complexes revealed a lateral binding to MR1, which was antigen (Ag)-independent and induced a weak TCR signaling.

These data indicated that rare T cells expressing a TCR γδ may interact with MR1 using an innate-like target recognition mechanism that is Ag-independent. However, these T cells’ physiological relevance and potential role in a pathological context remain unknown, as the described interactions induced no or weak T cell responses.

We investigated whether TCR γδ cells may also recognize MR1-Ag complexes by an adaptive-like mechanism. Among the different TCR γδ cell populations, we focused on those expressing TCR Vγ9Vδ2 heterodimers. In most healthy donors, the TCR Vγ9Vδ2 cell population represents the blood’s most abundant population (1-10% of total T cells). These cells react to phosphorylated isoprenoids (phospho-antigens, pAg) generated during isoprenoid synthesis in microbes and mammalian cells. The microbial isoprenoid biosynthesis pathway generates the pAg (E)-1-hydroxy-2-methyl-but-2-enyl 4-diphosphate (HMBPP) (15). In contrast, mammalian cells do not express this pathway and produce two pAgs, isopentenyl diphosphate (IPP) and dimethylallyl diphosphate (DMAPP) (16), intermediate products of the mevalonate pathway. The enzyme 3-hydroxy-3-methyl-glutaryl-coenzyme A reductase (HMGCR) is required for the synthesis of IPP and DMAPP, whereas the enzyme farnesyl pyrophosphate synthase (FPPS) allows their further utilization (17). The activation of this pathway occurs during the early phases of infections (18), thus contributing to the stimulation of TCR Vγ9Vδ2 cells by antigen-presenting cells (APCs) infected with microbes that do not produce HMBPP.

Different drugs acting on HMGCR and FPPS may directly modulate the accumulation or reduction of endogenous pAgs, thus controlling the stimulation of TCR Vγ9Vδ2 cells (16, 19). Statins block HMGCR, preventing the synthesis of the pAgs, and bisphosphonate drugs such as Zoledronate (Zol) block FPPS, leading to the accumulation of IPP and DMAPP. Therefore, modulating the mevalonate pathway directly affects the available pAgs.

The activation of TCR Vγ9Vδ2 cells by pAgs resembles the stimulation induced by innate receptors. Indeed, these cells are activated when butyrophilin 3A1 (BTN3A1) (20, 21) and butyrophilin 2A1 (BTN2A1) (22–24) are co-expressed by target cells. These BTNs trigger the TCR only in the presence of self (16) or exogenous pAgs (15, 25, 26). The presence of pAgs has two main functions, namely the generation of i) BTN3A homo and heterodimers formation, which is dependent on the juxtamembrane regions of the BTN3A chains, and ii) the interaction between the BTN2A1-B30.2 and BTN3A1-B30.2 domains (27), as recently reviewed (28). Without pAgs, BTN2A1 and BTN3A1 ectodomains block each other, and the TCR cannot be engaged (29). In contrast, in the presence of pAgs, which behave as molecular glues, they undergo conformational changes that promote TCR binding and productive T cell triggering (30, 31). More recent studies revealed that a supercomplex made of BTN2A1 homodimers and BTN3A1-3A2 heterodimers engage the TCR, with the BTN2A1 binding the Vγ9 chain and the BTN3A2 instead binding the Vδ2 chain (32).

Importantly, this type of cell activation does not require Ag presentation by classical antigen-presenting molecules and is defined as an innate-like mode of TCR stimulation (33, 34). Indeed, this activation does not involve TCR cognate interactions with Ag. It remains unknown whether some TCR Vγ9Vδ2 cells recognize Ags through an adaptive-like mechanism as observed in other T cells. Here, we show that MR1 promotes Ag-specific activation of a population of TCR Vγ9Vδ2 cells in a BTN-independent and CDR3δ-dependent manner, suggesting that they recognize MR1 like other MR1-restricted T cells (7, 11).

## Materials and methods

### Sex as a biological variable

Sex was not a variable considered in this study. The human primary data were pooled from both sexes. Our study also examined splenocytes from male and female animals; similar findings were reported for both sexes.

### Isolation of mouse TCR Vγ9Vδ2 tg cells

T cells were isolated from the spleen of TCR Vγ9Vδ2 tg and recombination-activating gene two (RAG-2)-deficient mice that express solely the human TCR Vγ9Vδ2 complexed with mouse CD3 (21). Mice were kept and bred at the animal facility of the Department of Biomedicine of the University Hospital Basel. Spleens were collected, and CD3^+^ cells were enriched using the MojoSort Mouse CD3 T cell isolation kit (Biolegend, #480023) according to manufacturer instructions. T cells were rested overnight in complete medium containing 10% FCS and used for activation assays.

Animal research was conducted under license 35328-2093, approved by the Authority of the Canton Basel-City.

### Tumor cell lines

The following tumor cell lines were purchased from the American Type Culture Collection (ATCC): A375 (human melanoma, CRL-1619, RRID: CVCL_0132) and HEK 293 T (human embryonic kidney, ACC 635, RRID: CVCL_0063). The following cell lines were previously generated in our laboratory: A375 β2m^−^, A375 β2m^−^ MR1, A375 β2m^−^ MR1 K43A (9, 35), J.RT3-T3.5, Jurkat-derived cells expressing a luciferase reporter gene under the control of NFAT, TCRαβ- and β2m-deficient (JKT) (36). Tumor cells were cultured in RPMI 1640 medium (Bioconcept, #1-41F01-I), 1mM sodium pyruvate (Bioconcept, #5-60F00-H), 1x non-essential amino acids (Bioconcept, #5-13K00-H), 1x stable glutamine (Bioconcept, #5-10K50-H), and 50 µg/ml of kanamycin (Bioconcept, #4-08F00-H) (complete medium) supplemented with 10% heat-inactivated fetal calf serum (FCS, BioConcept, #2-01F10-I). Cell lines were not authenticated and routinely confirmed to be absent of mycoplasma contamination by PCR.

### Knock-out cell lines generation

A375 β2m knock-out (A375 β2m^−^) cells were used to generate by CRISPR/Cas9 a BTN3A1-deficient cell line. For this purpose, A375 β2m^−^ cells were transduced with Lenti Cas9-Blast plasmid (Addgene, #52962) and selected for 10 days with 10 µg/ml Blasticidin (Gibco, #A1113903) before being transduced with lentiGuide-Puro (Addgene, #52963) with gRNAs targeting BTN3A1 (5’-CCAGAGGTGGATCGCCGCCC-3’ and 5’-GGCACTTACGAGATGCATAC-3’). After 96 h of selection with 2 µg/ml Puromycin (InvivoGen, #ant-pr-1), cell lines were generated from single-cell clones by limiting dilution. DNA was extracted by each cell clone with a Macherey-Nagel Tissue DNA extraction kit (#740952.250) and BTN3A1 gRNA target locus was amplified with Q5 polymerase (NEB, #M0492S) and the following primers: BTN3A1_For 5’-TCCTCTGAGATTTTAGCATGAG-3’ and BTN3A1_Rev 5’-TGGCAATGACTAGGAATTGG-3’. PCR products were sequenced using the Sanger method, and effective BTN3A1 gene inactivation was confirmed by comparing the PCR product with the wild-type BTN3A1 sequence using Benchling [Biology Software (2023)].

### T cell lines and clones

PBMCs were isolated by density-gradient centrifugation using Lymphoprep (Stemcell, #07851). The used T cell clones were already established in our laboratory or generated from bulk lines, cultured, and expanded as described in (11, 21). *Ex vivo* TCR γδ cells were negatively enriched from PBMCs using EasySep™ Human Gamma/Delta T Cell Isolation Kit (Stemcell, #19255) and used for proliferation and activation assays. TCR Vγ9Vδ2 cells were FACS-sorted from PBMCs using anti-Vδ2 (Clone B6; Biolegend) and anti-Vγ9 (Clone B3; Biolegend) mAbs. T cells were cultured in a complete medium supplemented with 5% human AB Serum (Blood Donation Center of the University Hospital of Basel) and 100 U/ml recombinant human IL-2 (Peprotech, #200-02).

### TCR gene transfer

RNA from T cell clones was extracted with Nucleospin RNA (Macherey-Nagel, #740955.50), and cDNA was synthesized with SuperScript III reverse transcriptase (Invitrogen, #18080093). TCR Vγ9 and Vδ2 chain transcripts were amplified with gene-specific primers, followed by Sanger sequencing and respective analysis using ImMunoGeneTics (http://www.imgt.org). The transcripts were amplified using primers containing cloning adaptors and cloned into a lentiviral vector (Addgene, #52962) using the In-Fusion HD Cloning Kit (Takara, #639649). The vectors containing TCR Vγ9 or Vδ2 sequences and the lentivirus packaging plasmids pMD2.G (Addgene, #12259), pMDLg/pRRE (Addgene, #12251), pRSV-REV (Addgene, #12253) and the pAdVAntage (Promega, #E1711), were co-transfected into HEK 293 T LX cells using Metafectene PRO reagent (Biontex, #T040-1.0). JKT cells were transduced with lentiviral supernatants containing TCR Vγ9 and Vδ2 sequences. JKT cells expressing the different TCRs were sorted based on CD3 surface expression and used for functional assays.

### Cell surface MR1 upregulation

A375 cells were tested for MR1 surface expression by flow cytometry. Briefly, cells were plated at 10^5^/well and incubated with 50 µM Zol (Sigma-Aldrich, # SML0223-50MG), 30 µM Ac-6-FP (Schircks Laboratory, #11.418), or vehicle (PBS) for 4 h at 37°C. After incubation, cells were first blocked for 15 min at 4°C for unspecific binding using 50% human AB serum, 0.1% BSA, 0.02% NaN_3_ in PBS, and then stained with anti-human MR1 mAbs (clone 26.5; Biolegend, #361108) for 20 min at 4°C. Cells were washed and resuspended in PBS with 0.5 μg/ml 4′,6-diamidino-2-phenylindole (DAPI, Sigma-Aldrich, #MBD0015). Flow cytometry analysis was performed on a Cytoflex (Beckman Coulter).

### Analysis of MR1 gene expression

A375 WT and A375 β2m^−^ MR1 cells were exposed to 30 µM Ac-6-FP, 50 µM Zol, or vehicle (PBS) for 4 h at 37°C in complete medium supplemented with 10% FCS. RNA was isolated from 10^6^ cells with NucleoSpin RNA Mini Kit (Macherey-Nagel, # 740955) following the manufacturer’s instructions, and 500 ng of RNA were used to synthesize cDNA with the PrimeScript Reverse Transcriptase kit (Takara, #2680Q). Quantitative PCR reactions were performed with Power Sybr green PCR master mix (Applied Biosystems, #4367659) using 15 ng of cDNA per reaction with the following primers for MR1 (forward: 5’-TGGCAGCAGATGTTCAAGGTGG-3’, reverse: 5’-GAAATCCTGTGGTGCTTCCATCC-3’) and using ribosomal protein S18 (RPS18) as housekeeping gene (forward 5’-GCAGAATCCACGCCAGTACAAG-3’, reverse 5’-GCTTGTTGTCCAGACCATTGGC-3’). MR1 relative expression was calculated using the ΔΔCt method (37).

### Generation of soluble MR1 protein

Soluble recombinant human β2m-MR1 Fc-Streptag and β2m-MR1 K43A Fc-Streptag (an MR1 mutant form with lysine in position 43 mutated into an alanine) were prepared as described (11). Briefly, A375 cells secreting the recombinant proteins were cultured for 50 h, then the supernatant was collected. Soluble MR1 was quantified by ELISA as described (11) and stored at 4°C until further use to coat magnetic beads.

A second form of soluble MR1 protein was generated by assembling in the bacterial expression vector pET23d(+) DNA-Novagen (Sigma-Aldrich, #69748-M) a hybrid construct containing the nucleotide sequences coding for β2m, followed by a sequence encoding a (G_3_S)_3_ flexible linker and the soluble portion of MR1 (MR1*01 allele, GenBank accession number NM_001531). As described, the protein was produced in bacteria as inclusion bodies, refolded, and purified without ligand addition (11). This molecule was used in Fluorescence polarization assays. The same protein was also refolded in the presence of the following ligands: Ac-6-FP, (2E)-3-({9-[3,4-dihydroxy-5-(hydroxymethyl)oxolan-2-yl]-9H-purin-6-yl}amino)prop-2-enal (M_1_Ado), (2E)-3-({9-[4-hydroxy-5-(hydroxymethyl)oxolan-2-yl]-9H-purin-6-yl}amino) prop-2-enal (M_1_dA) and 3H,10H-pyrimido[1,2-a]purin-10-one (M_1_Gua) as described (11). These MR1-bearing ligand monomers were used to generate MR1 tetramers.

### Preparation of soluble MR1-coated beads

MagStrep “type 3” XT Beads (IBA, #2-4090-010) were washed with PBS and incubated with 30 µg/ml of Streptag-soluble MR1 at 37.5 pg/bead for 18 h at 4°C under shaking conditions. Control beads were incubated with supernatant derived from A375 cells, which did not produce soluble MR1. This group is indicated as MR1-negative beads. Then, MR1-coated beads were washed with PBS and used for activation assays or TCR tetramer staining experiments.

### T cell activation assay

Activation assays were done in 384-well plates, co-culturing human or mouse T cells (2 x 10^4^/well) with A375 (4 x 10^4^ cells/well). Human T cells were also cultured with MR1-coated beads (8 x 10^4^ cells/well) for 18 h. All assays were performed in a final volume of 80 μl. T cell activation was evaluated after 18 h by measuring human IFN-γ released in the supernatant by ELISA and measuring surface expression of CD69, CD25, and CD137 markers by FACS. In some cases, proliferation and activation assays were performed in the presence of anti-human MR1 mAbs (clone 26.5; Biolegend, #361102), anti-human HLA-A, B, C mAbs (clone W6/32; Biolegend, #311427), or IgG2a isotype-matched mAbs (clone MOPC-173; Biolegend, #400202), all at 30 µg/mL.

JKT activation assays were performed in an opaque 384-well plate (Corning, #3570) (3 x 10^4^ T cells/well and 3 x 10^4^ A375 cells/well) for 8 h.

APCs were treated with Zol (50 µM), Ac-6-FP (30 µM), IPP (10 µM) (Sigma-Aldrich, #I0503) or 5-OP-RU (40 nM) for 4 h before co-culture with T cells or JKT cells. In some cases, APCs were incubated with agonist anti-BTN3A mAbs (clone 20.1, Invitrogen, #14-2779-82) at 1 µg/mL for 1 h before adding JKT cells. Competition assays were performed by incubating APCs with Ac-6-FP (30 µM) for 2 h before Zol incubation. Luciferase production was assessed using the Bio-Glo kit (Promega, #G7940) according to the manufacturer’s protocol, and luminescence was measured using Sinergy H1 (Hybrid Reader, Biotek).

For intracellular cytokine measurement, T cells were treated with 2 µM Monensin (Biolegend, #420701) and 20 µg/ml Brefeldin A (Biolegend, #420601) before being challenged for 12 h with MR1-coated beads or PMA (50 ng/ml, Sigma-Aldrich, #P1585) and Ionomycin (500 ng/ml, Sigma-Aldrich, #I0634).

### Cytokine analysis

IFN-γ release was measured by ELISA as previously described (9): human IFN-γ (Biolegend, capture MD-1 mAb, #507502; revealing biotinylated 4S.B3 mAb, #502504), HRP Streptavidin (Biolegend, #405210), and recombinant human IFN-γ (Peprotech, #300-02).

### Soluble TCR production and tetramerization

D9A10 TCR soluble ectodomains were expressed in *E. coli* as inclusion bodies, folded *in vitro*, and purified as described below. The D9A10 Vδ and Vγ genes were fused to the ones of Cα and Cβ, respectively, for the production of soluble TCR γδ ectodomains (14, 38). As described, a disulfide bond was engineered between the Cα and Cβ chains (39) to facilitate the formation of a stable heterodimeric complex. The Vγ/Cβ chain was engineered with a C-terminal AviTag. The resulting chimeric TCR chains were codon optimized for *E. coli* expression and separately cloned into the vector pET23d(+) DNA-Novagen (Sigma-Aldrich, #69748-M). Inclusion bodies of both chains were produced in *E. coli* BL21(DE3) pLysS (Thermo Fisher, #C606010). Vδ/Cα and Vγ/Cβ inclusion bodies (15 mg, each) were solubilized at 10 mg/ml in 6 M guanidine HCl, 50 mM β-mercaptoethanol, 10 mM EDTA, 50 mM Tris pH 8.1 and heated at 50°C for 30 min, under shaking. Insoluble debris were removed by centrifugation at 20,000 RCF for 10 min. D9A10 soluble monomers were folded *in vitro* by rapid dilution of the solubilized inclusion bodies in 500 ml refolding buffer consisting of 5 M urea, 0.4 M L‐arginine, 100 mM Tris pH 8.1, 3.7 mM oxidized glutathione, 6.6 mM reduced glutathione to reach a final concentration of to 60 mg/l. Folded monomers were purified by two ion exchange chromatography steps with HiTrap DEAE FF (Cytiva, #17515401) and Mono Q™ 5/50 GL (Cytiva, #17516601) columns. Elution fractions were analyzed by SDS-PAGE using reducing and non-reducing conditions to confirm the disulfide bond formation between the two chains. The TCR-containing fractions were pooled and biotinylated *in vitro* with BirA biotin-protein ligase bulk reaction kit (Avidity, Bulk BirA) and purified by size exclusion chromatography using a Superdex 75 10/300 GL column (Cytiva, #17517401).

### MR1 and TCR tetramerization

Biotinylated TCR and Ag-loaded MR1 monomers were tetramerized using PE-conjugated streptavidin (Prozyme, catalog no. PJRS25) at a 4:1 molar ratio. For each biotinylated monomer (5µg), we added 8µg of PE-conjugated streptavidin. PE-conjugated streptavidin was prepared at 200 µg/mL in PBS and 1.6 µg were added stepwise to the biotinylated monomer in cold and dark conditions every 20 min under shaking.

### Flow cytometry

Details of mAbs used can be found in **Supplementary Table S1**. All mAbs were titrated to identify optimal concentrations before their use in multicolor flow cytometry. Cells were stained with Fixable Viability Kits as Zombie NIR (Biolegend, #423106), Zombie AQUA (Biolegend, #423101), or LIVE/DEAD Fixable Blue (Thermo Fisher, #L23105) in PBS for 20 min at 4°C, followed by surface staining by adding specific mAbs for 20 min in PBS, 0.5% BSA, 0.02% NaN_3_ at 4°C. When mentioned, a viability assessment was done by adding DAPI after cell surface staining.

For intracellular staining, stained cells were fixed with a Fixation Buffer (Biolegend, #420801) for 15 min at RT. Cells were permeabilized using Intracellular Staining Permeabilization Wash Buffer 1X (Biolegend, #421002) for 20 min at 4°C, followed by anti-human mAbs.

Cells were first gated based on forward scatter-area (FSC-A) and side scatter-area (SSC-A), followed by gating on viable cells. Singlets were gated using SSC-A/side scatter height (SSC-H) and FSC-A/forward scatter height (FSC-H). Cells were acquired by an Aurora spectral analyzer (Cytek) or CytoFLEX flow cytometer (Beckman Coulter) and analyzed using FlowJo v10 software (LLC). TCR Vδ2 cells were sorted using a FACSMelody Cell Sorter (BD Biosciences).

MR1-coated beads were stained with D9A10 TCR tetramer (5 µg/ml) for 20 min at room temperature in PBS, followed by the addition of anti-PE Abs from the PE-positive Selection Kit (Stemcell, #17684) for an additional 20 min. Beads were washed with PBS and acquired using a CytoFLEX flow cytometer (Beckman Coulter).

### Fluorescence polarization for MR1-binding assay

Soluble recombinant single-chain MR1 (80 nM) was incubated for 18 h at room temperature with 25 nM of the reporter fluorochrome JYM20 (Wuxi, #WX10-01-245) and Zol or Ac-6-FP at different concentrations in fluorescence polarization assay buffer (20 mM TRIS pH 8.5, 2 mM EDTA, 150 mM NaCl, 0.05% Tween 20). Samples in 96 well plates (Corning, #3686) were read using a Spark multimode microplate reader (Tecan; Excitation wavelength: 535nM. Emission wavelength 595nm. Temperature 25°C).

### Bioinformatic analysis

Flow cytometry data was exported from FlowJo v10.7.1 and imported in R v4.2.0 through the Bioconductor package flowCore v2.10.0 (40), and clustering analysis was performed. Marker expression levels were transformed using the inverse hyperbolic sine transformation (asinh function within R) with a cofactor of 150. Clusters were computed via the R implementation of Phenograph (41), considering the transformed expression values for 9 markers (TCR Vδ2, CD69, CD26, CD28, CD56, CD57, CD161, NKp80, CD95) and a k of 100. A heatmap of the average expression of each marker in each cluster was produced using pheatmap v1.0.12 (42), and the clusters were visualized as colors overlaid on a Uniform Manifold Approximation and Projection (UMAP) using the R implementation of UMAP (v0.2.10.0) (43).

### Human samples and study approval

Blood samples from healthy donors and the investigated patient were obtained from the University Hospital Basel. The research with healthy blood samples and the prospective cohort study of the functional and genetic architecture of patients with primary immune dysregulation (FuGe-PID) have been approved by the Ethics Committee North-West & Central Switzerland (EKNZ 2017-01888 and EKNZ215-187).

All donors consented in writing to analyze their samples.

### Statistical analysis and data visualization

Statistical analyses and data visualization were performed using GraphPad Prism v10 (GraphPad Software, Inc.). P values are indicated in the figure panels and legends.

The assembling of figure panels was done using Affinity Designer 2.5.3 (Serif Europe).

## Results

### TCR Vγ9Vδ2 cells react to MR1

**Figure 1:**
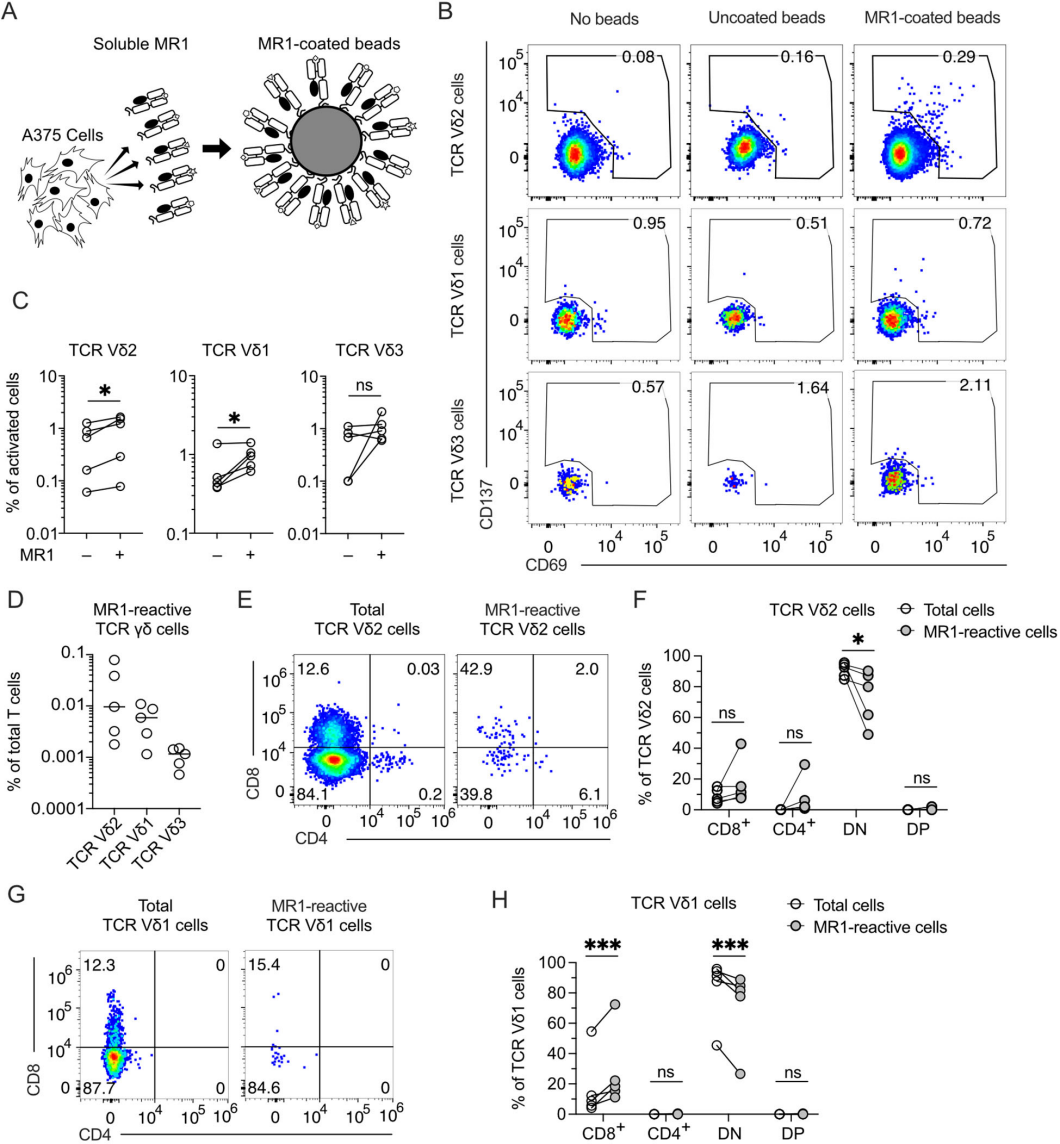
MR1-reactive TCR γδ cells are present in the peripheral blood of healthy donors. **(A)** Schematic representation of beads coated with a soluble form of MR1 secreted by A375 cells. MR1-coated beads were used to stimulate TCR γδ cells negatively enriched from PBMCs of healthy donors. **(B)** Activation of enriched TCR γδ cells freshly isolated from PBMCs upon challenge with MR1-coated or uncoated beads. Flow cytometry plots show the expression of CD69 (x-axis) and CD137 (y-axis) on TCR Vδ2-, Vδ1-, and Vδ3-expressing cells. Numbers indicate percentages of cells in the gated areas. Data is representative of one healthy donor among the five investigated. **(C)** Summary of activated TCR Vδ2-, Vδ1-, and Vδ3-expressing cells after stimulation with MR1-coated or uncoated beads. Each symbol represents a different donor. Two-tailed ratio paired t-test. ^∗^ *p* < 0.05; ns, not significant. **(D)** Summary of MR1-reactive TCR Vδ2-, Vδ1-, and Vδ3-expressing cells as a percentage of total T cells. Each symbol represents a different donor. Bars indicate the median values. **(E, G)** CD4 and CD8 expression on total **(E)** TCR Vδ2 cells or **(G)** TCR Vδ1 cells and on MR1-reactive **(E)** TCR Vδ2 cells or **(G)** TCR Vδ1 cells. Numbers indicate percentages of cells in the quadrants. Data is representative of one healthy donor among the five investigated. **(F, H)** Summary of CD4 and CD8 expression on total **(F)** TCR Vδ2 cells or **(H)** TCR Vδ1 cells and on MR1-reactive **(F)** TCR Vδ2 cells or **(H)** TCR Vδ1 cells. Each dot represents a different donor. DN, double negative; DP, double positive. Two-way ANOVA with repeated measures, Sidak’s multiple comparisons test. ns, not significant; ^∗^ *p* < 0.05; ^∗∗∗^ *p* < 0.001; ns, not significant.

We systematically analyzed whether TCR γδ cells react to MR1 and are present in healthy donors. To exclude the contribution of molecules other than MR1 to this reactivity, we established a new assay using beads coated with soluble MR1, produced by the melanoma cell line A375, as stimulatory reagents (**Figure 1A**; **Supplementary Figures 1A, B**). The purity of the MR1 bound to the beads was confirmed by SDS-PAGE (**Supplementary Figure 1C**). Control beads were incubated with supernatant derived from A375 cells that do not produce soluble MR1. The activation assays were performed using TCR γδ cells purified from peripheral blood (**Supplementary Figure 1D**) in the presence of MR1-coated beads without any cytokine or other cell types to exclude the participation of molecules besides MR1 in the activation. The MR1-reactive T cells were detected by co-staining with anti-CD69, anti-CD137, and anti-TCR Vδ mAbs (**Figure 1B**) in five healthy donors (**Figure 1C**). We found that rare TCR γδ cells were activated using these stringent conditions, with those expressing the TCR Vδ2 and Vδ1 chains being the most abundant, whereas MR1-reactive TCR Vδ3 cells were much less frequent or even absent (**Figures 1C, D**).

Among the MR1-reactive TCR Vδ2 cells, CD4 and CD8-double negative (DN) were reduced, and in some donors, CD4^+^ or CD8^+^ cells were increased (**Figures 1E, F**). MR1-reactive TCR Vδ1 cells showed reduced CD4-CD8 DN and increased CD8^+^ cells (**Figures 1G, H**). These findings revealed the existence of MR1 self-reactive TCR Vδ2 cells and confirmed the existence of MR1-interacting TCR Vδ1 and TCR Vδ3 cells (12, 14). We focused on Vγ9Vδ2 cells, which represent the majority of the circulating TCR γδ cells.

**Figure 2:**
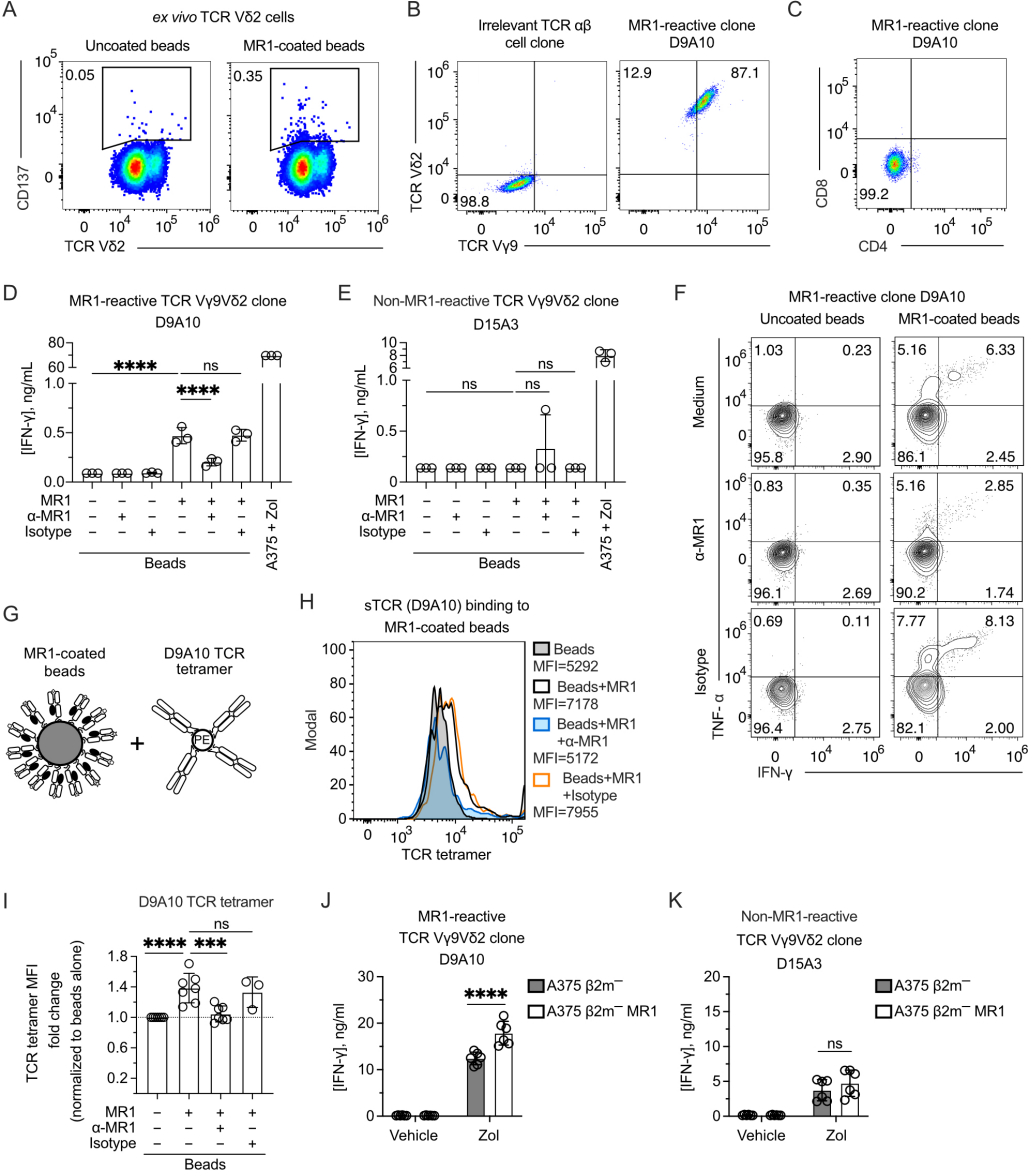
The TCR Vγ9Vδ2 cell clone D9A10 interacts with MR1 and is activated upon MR1 recognition. **(A)** Gating used to sort activated TCR Vδ2 cells from PBMCs upon challenge with MR1-coated or uncoated beads. Flow cytometry plots show the expression of TCR Vδ2 and CD137. Numbers indicate percentages of cells in the gated areas. **(B)** Staining with anti-Vγ9 and anti-Vδ2 mAbs on an irrelevant TCR αβ cell clone (left) and on the MR1-reactive cell clone D9A10 (right). Numbers indicate percentages of cells in the quadrants. **(C)** CD4 and CD8 phenotype of the T cell clone D9A10. **(D, E)** Activation of **(D)** D9A10 and **(E)** D15A3 cell clones using beads coated or not with soluble MR1 in the presence or absence of anti-MR1 or isotype-matched mAbs. Both clones were stimulated with A375 cells treated with Zol (10µM) as a positive control. Data is representative of three independent experiments. Bar plots of IFN-γ release mean ± SD of triplicate independent cultures. One-way ANOVA, Dunnett’s multiple comparisons test. ^∗∗∗∗^ *p*< 0.0001; ns, not significant. **(F)** Intracellular staining of IFN-γ and TNF-α in D9A10 clone after stimulation using beads coated or not with soluble MR1, in the presence or absence of anti-MR1 or isotype-matched mAbs. Numbers indicate percentages of cells in the quadrants. Data is representative of three independent experiments. **(G)** Schematic representation of the reagents used for staining MR1-coated beads with D9A10 TCR tetramers. **(H)** Staining of uncoated- or MR1-coated beads using D9A10 TCR tetramers in the presence or absence of anti-MR1 or isotype-matched mAbs. Histograms are representative of at least three independent experiments. Median fluorescence intensity (MFI) is indicated next to each histogram. **(I)** Summary of D9A10 TCR tetramer staining of MR1-coated or uncoated beads in the absence or presence of anti-MR1 or isotype-matched mAbs. TCR tetramer MFI was normalized to uncoated beads. Bar plot of mean ± SD of at least three independent experiments. Each dot represents an independent experiment. One-way ANOVA, Dunnet’s multiple comparisons test. ^∗∗∗^ *p*< 0.001; ^∗∗∗∗^ *p*< 0.0001; ns, not significant. **(J, K)** Activation of the TCR Vγ9Vδ2 clones **(J)** D9A10 and **(K)** D15A3 after stimulation with A375 β2m^−^ cells (filled bars) or A375 β2m^−^ MR1 cells (open bars) pulsed with Zol or vehicle. Bar plots of IFN-γ release mean ± SD of triplicate independent cultures of two independent experiments. Two-way ANOVA, Sidak’s multiple comparisons test. ^∗∗∗∗^ *p* < 0.0001; ns, not significant.

The TCR Vγ9Vδ2 cell reactivity to MR1 was validated by sorting and cloning the cells that upregulated CD137 upon stimulation with MR1-coated beads (**Figure 2A**). The isolated clones expressed Vγ9 and Vδ2 chains, as represented by the D9A10 clone (**Figure 2B**). This clone was CD4-CD8 DN (**Figure 2C**; **Supplementary Figure 2A**) and released IFN-γ (**Figure 2D**) when challenged with MR1-coated beads (**Supplementary Figures 2B, C**). This reactivity was blocked by adding anti-MR1 antibodies and not isotype-matched ones (**Figure 2D**). In contrast, a control Vγ9Vδ2 clone (D15A3) was not MR1-reactive but was highly reactive to APC pulsed with Zol (**Figure 2E**). The response of D9A10 cells to MR1-coated beads was also observed using intracellular detection of TNF-α and IFN-γ (**Figure 2F**; **Supplementary Figures 2D–F**). Instead, the D15A3 cells did not produce these cytokines (**Supplementary Figure 2E**). Both T cell clones produced TNF-α and IFN-γ upon stimulation with A375 cells pulsed with Zol (**Supplementary Figure 2F**).

To further address the interaction between MR1 and the D9A10 TCR, we generated a soluble tetramerized D9A10 TCR to stain MR1-coated beads (**Figure 2G**). The purity of the sTCR was confirmed by SDS-Page and size-exclusion chromatography (**Supplementary Figures 2G, H**). This reagent bound to MR1-coated beads but not the uncoated ones, and the binding was prevented using anti-MR1 antibodies but not isotype-matched ones (**Figure 2H**). Similar results were observed in at least three independent experiments performed using different preparations of soluble MR1 (**Figure 2I**).

Further experiments investigated whether D9A10 cells reacted to β2m-deficient APC lacking or expressing MR1. We previously described the generation of A375 cells deficient in β2m (A375 β2m−) and transduced with the MR1 gene (A375 β2m− MR1) encoding the MR1*01 allele (9). These cells are positive for MR1 but not stained with an antibody reacting to all HLA A, B, and C molecules (**Supplementary Figure 2I**). The D9A10 clone did not react to A375 β2m− and A375 β2m− MR1 cells (**Figure 2J**), although it responded to MR1-coated beads (**Figure 2D**). This discrepancy could be caused by a small number of MR1-Ag complexes expressed on APC compared to beads. When the APCs were pulsed with Zol, unexpectedly, the MR1-positive APCs were more stimulatory than the MR1-negative ones (**Figure 2J**). In contrast, the D15A3 cells, which are MR1 non-reactive, were equally stimulated by Zol-pulsed APCs regardless of MR1 expression (**Figure 2K**). These findings confirmed the existence of MR1-reactive Vγ9Vδ2 TCRs. Furthermore, the MR1-reactive TCRs could increase the response to Zol-treated APCs expressing MR1.

### High frequency of MR1-self-reactive TCR Vγ9Vδ2 cells in a patient with autoimmunity and TCR γδ lymphocytosis

**Figure 3:**
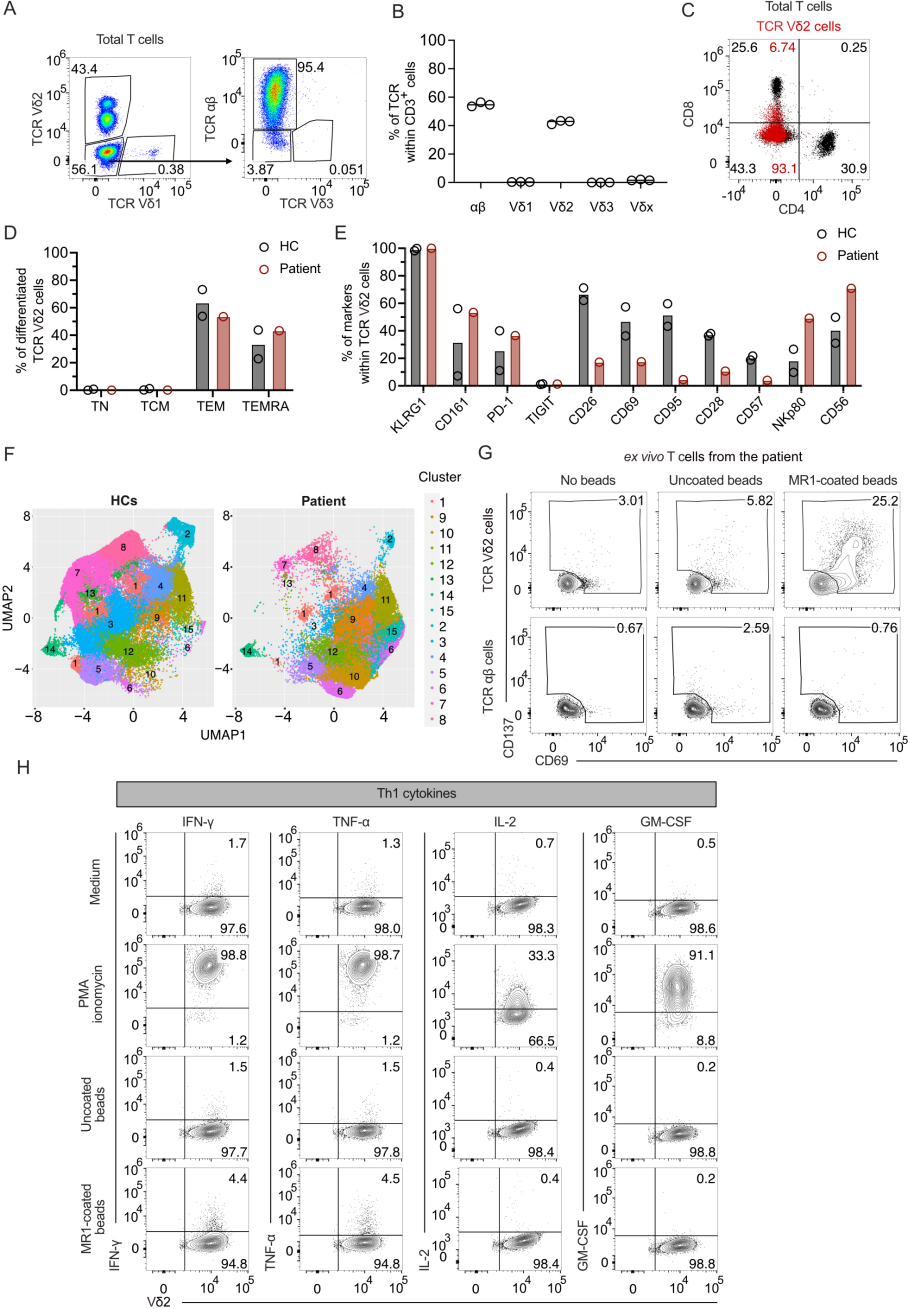
MR1-reactive TCR Vγ9Vδ2 cells are increased in a patient with TCR γδ cell lymphocytosis. **(A)** Characterization of T cell populations derived from patient’s PBMCs. Flow cytometry plots of TCR Vδ1 *vs*. TCR Vδ2 expression on total T cells (left panel) and TCR Vδ3 *vs*. TCR αβ expression on TCR Vδ1^−^/ Vδ2^−^ cells (right panel). Numbers indicate the percentage of cells in the gated areas. **(B)** Percentages of the T cell populations based on TCR aβ and Vδ expression by flow cytometry. Each dot represents a technical replicate. The bar is the median of the independent measurements. **(C)** CD4 and CD8 expression on total T cells (black dots) or total TCR Vδ2 cells (superimposed red dots). Numbers indicate CD4 and CD8 cell percentages in each quadrant according to the color. **(D)** Percentages of naïve (TN), central memory (TCM), effector memory (TEM), and effector memory RA^+^ (TEMRA) T cells in TCR Vδ2 cells from PBMCs of two healthy controls (HC) and the patient (red). Each dot represents a different donor. **(E)** Expression of the markers KLRG1, CD161, PD-1, TIGIT, CD26, CD69, CD95, CD28, CD57, NKp80, and CD56 on PBMC-derived TCR Vδ2 cells from HCs (black) and patient (red). Each dot represents a different donor. **(F)** UMAP of TCR Vδ2 cells from two HCs and the patient distributed in clusters 1 to 15 according to TCR Vδ2, CD161, CD26, CD69, CD95, CD28, CD57, NKp80, and CD56 expression. **(G)** Activation of *ex vivo* patient’s PBMC-isolated T cells challenged with MR1-coated or uncoated beads. Flow cytometry plots of CD69 and CD137 expression on TCR Vδ2-gated cells (top plots) or TCR αβ-gated cells (bottom plots). Numbers indicate the percentage of positive cells in the gated area. **(H)** Activation of the patient-derived TCR γδ cells stimulated or not with PMA/ionomycin and with MR1-coated or uncoated beads. Flow cytometry plots of intracellular IFN-γ, TNF-α, IL-2, or GM-CSF (y-axis) and surface TCR Vδ2 (x-axis).

To study the relevance of MR1-autoreactive TCR Vγ9Vδ2 cells, we examined one patient with abnormal expansion of TCR γδ cells, who reported recurring infections of unknown origin, manifesting fever, chills, and swelling of inguinal and cervical lymph nodes. The patient suffers from atopy and clinically relevant type I sensitization to house dust mites, tree, and grass pollen; however, at hospital admission, the patient had normal IgE antibody levels (**Supplementary Table 2**). The hemogram reported no alterations in leukocyte absolute numbers or other types of circulating blood cells (**Supplementary Table 3**), except for imbalanced T cell populations, with abnormally increased numbers of DN T cells and low CD4^+^ T cells (**Supplementary Table 4**). In the past five years, the patient suffered sudden increases in absolute lymphocyte numbers, particularly of DN T cells, during symptomatic exacerbations of infections (**Supplementary Table 4**). Flow cytometry studies were performed to investigate whether the lymphocytosis and the increased numbers of DN cells were associated with the expansion of unconventional T cells. An increased frequency of TCR Vδ2 cells was detected, which comprised ~43.4% of total T cells in the blood, whereas TCR αβ cells were ~53.6% (**Figure 3A**). TCR Vδ1 cells were ~0.4% of total T cells, and TCR γδ cells expressing other TCR Vδ chains were <2.2% of total T cells (**Figure 3A**). These findings were confirmed in three independent analyses (**Figure 3B**). Among circulating T cells, CD4^+^ cells were 30.9%, CD8^+^ cells were 25.6%, and DN cells were 43.3%. TCR Vδ2-expressing cells were mostly DN (93.1% of total TCR γδ cells), and only 6.7% expressed dull CD8 levels (**Figure 3C**).

We performed a multicolor flow cytometry analysis of 15 surface markers on the patient’s peripheral blood mononuclear cells (PBMCs) to assess the functional maturation of circulating T cells. Two healthy donors were investigated in parallel as controls (HC). Almost all TCR Vδ2 cells from the patient and the HCs were Ag-experienced. In all three donors, ~60.0% of cells showed the phenotype of T effector memory (T_EM_) cells, and ~40.0% had a T effector terminally differentiated (T_EMRA_) profile (**Figure 3D**). The cells from the patient and HCs expressed high levels of KLRG1, further emphasizing that these cells were similarly differentiated. Other markers, including CD161 and PD-1, were also equally expressed, whereas TIGIT was not expressed (**Figure 3E**).

The activation markers, CD26 and CD69, the Fas receptor CD95, and the co-stimulatory molecule CD28 were reduced in the patient compared to the HCs, whereas the NK receptors NKp80 and CD56 were increased (**Figure 3E**). Cluster analysis considering differently expressed markers showed the main dissimilarities between TCR Vδ2 cells from the patient and HCs (**Figure 3F**). Four cell populations (clusters 6, 9, 10, and 15) were more frequent in the patient sample than in controls (**Supplementary Figure 3A**). The cells in these clusters were enriched in those co-expressing NKp80, CD161, CD56; markers shared with NK cells (44); and CD26. In contrast, clusters 1, 2, 3, 4, 7, 8, and 13 were more frequent in HCs. These latter clusters expressed reduced or absent NKp80, CD161, and CD56. They were variably positive for CD26 and CD95 (**Supplementary Figure 3A**), indicating the presence of cell populations with different degrees of differentiation after stimulation.

We next investigated the frequency of T cells in the patient that were MR1-reactive. The T cells freshly isolated from patient-derived PBMCs stimulated with MR1-coated beads, as previously described (**Figure 1A**), upregulated the activation markers CD69 and CD137 in ~25.2% of the Vδ2 cells (**Figure 3G**). In control cultures with T cells alone and T cells cultured with uncoated beads, ~3.0% and 5.8% of cells showed background activations. In contrast, TCR αβ cells did not upregulate CD69 or CD137 under the same conditions (**Figure 3G**). Thus, a significant fraction of TCR Vδ2 cells were MR1-autoreactive.

To determine the functional phenotype of the MR1-reactive TCR Vδ2 cells, we measured the intracellular cytokine profile of a patient-derived TCR Vδ2 cell line upon activation. PMA/Ionomycin stimulation revealed the production of mainly Th1 cytokines (IFN-γ, TNF-α, IL-2, and GM-CSF) and IL-13 but not IL-4, IL-10, or IL-17A (**Figure 3H**; **Supplementary Figure 3B**). When T cells were incubated with MR1-coated beads, 4.4% produced IFN-γ and 4.5% TNF-α. Other cytokines were undetected (**Figure 3H**; **Supplementary Figure 3B**). As a control, the same TCR Vδ2 cells incubated with uncoated beads did not produce cytokines (**Figure 3H**; **Supplementary Figure 3B**). Thus, the MR1-reactive TCR Vδ2 cells derived from this patient produced type 1 cytokines and showed a Th1 pro-inflammatory functional profile.

### MR1-self-reactive Vγ9Vδ2 cells recognize MR1-expressing APCs

Next, we studied the MR1 recognition mechanism of TCR Vγ9Vδ2 cells and examined the relevance of MR1, TCR, BTN3A1, Ag, and the Vδ2 chain.

**Figure 4:**
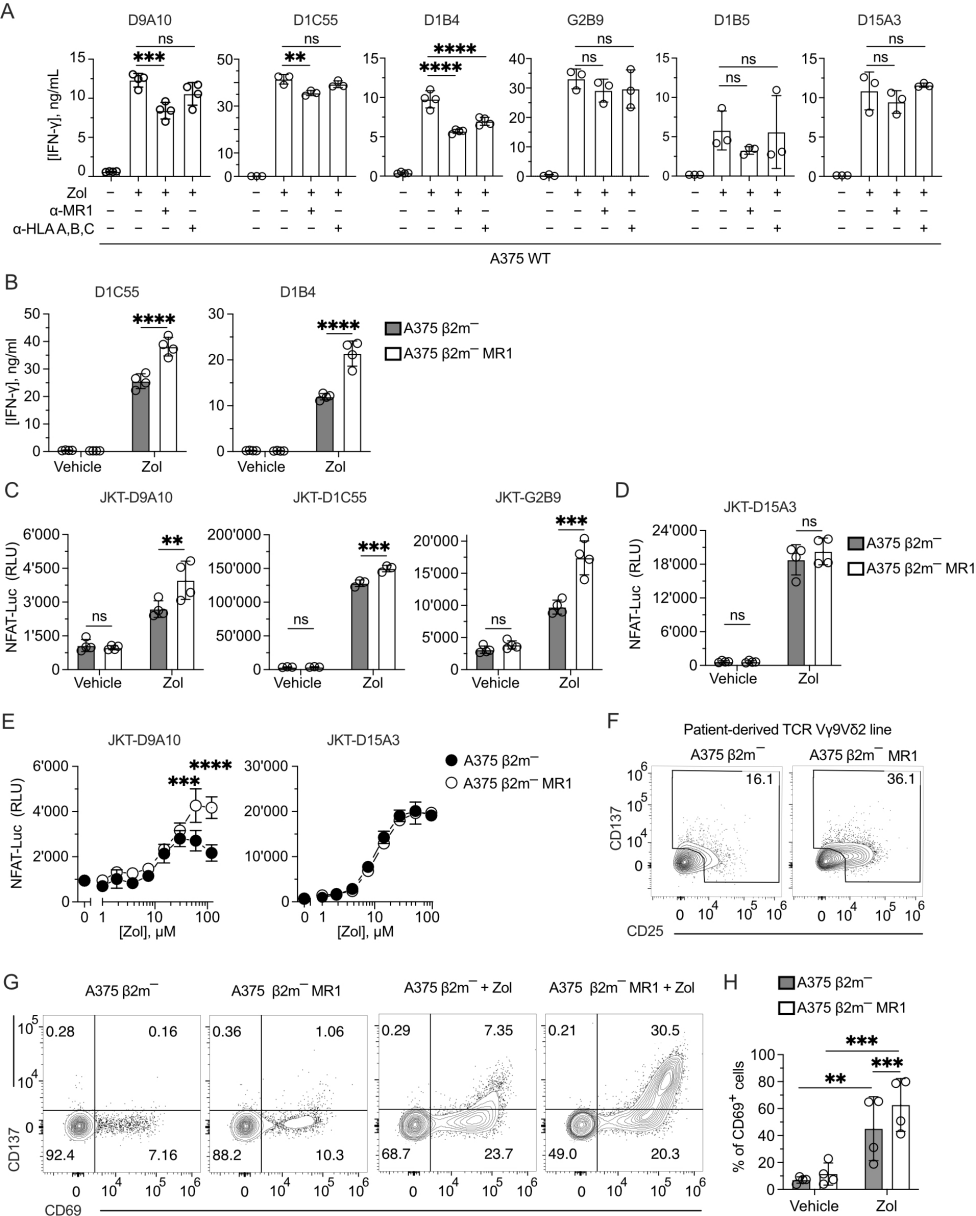
MR1 blocking reduces TCR Vγ9Vδ2 cell activation, and TCR Vγ9Vδ2 transfer recapitulates MR1 recognition. **(A)** Response of the indicated TCR Vγ9Vδ2 cell clones to A375 WT pulsed with Zol for 4 h in the presence or absence of anti-MR1 or anti-HLA A, B, C mAbs. Data is representative of three independent experiments. Bar plots of IFN-γ release mean ± SD of at least triplicate independent cultures. One-way ANOVA, Dunnet’s multiple comparisons test. ^∗∗^ *p* < 0.01; ^∗∗∗^ *p* < 0.001; ^∗∗∗∗^ *p* < 0.0001; ns, not significant. **(B)** Response of the TCR Vγ9Vδ2 clones D1C55, and D1B4 when stimulated with A375 β2m^−^ cells (black bars) and A375 β2m^−^ MR1 cells (white bars) pulsed with Zol. Data is representative of three independent experiments. IFN-γ measurement is the mean ± SD of quadruplicate independent cultures. Two-way ANOVA, Sidak’s multiple comparisons test. ^∗∗∗∗^ *p* < 0.0001; ns, not significant. **(C, D)** Activation of JKT cells expressing **(C)** MR1-reactive TCRs Vγ9Vδ2 (D9A10, D1C55, G2B9) and **(D)** the non-MR1-reactive TCRs Vγ9Vδ2 (D15A3) challenged with A375 β2m^−^ cells or A375 β2m^−^ MR1 cells exposed to Zol or vehicle. Data is representative of three independent experiments. Plots show the RLU mean ± SD of at least triplicate independent cultures. Two-way ANOVA, Sidak’s multiple comparisons test. ^∗∗^ *p*< 0.01; ^∗∗∗^ *p* < 0.001; ^∗∗∗∗^ *p* < 0.0001; ns, not significant. **(E)** Activation of JKT cells expressing the MR1-reactive TCR Vγ9Vδ2 (D9A10) and non-MR1-reactive TCRs Vγ9Vδ2 (D15A3) challenged with A375 β2m^−^ cells (black dots) or A375 β2m^−^ MR1 cells (white dots) exposed to increasing doses of Zol. Data is representative of three independent experiments. Plots show the RLU mean ± SD of triplicate independent cultures. Two-way ANOVA, Sidak’s multiple comparisons test. ^∗∗∗^ *p* < 0.001; ^∗∗∗∗^ *p* < 0.0001. **(F)** Activation of the patient-derived TCR γδ cell line stimulated with untreated A375 β2m^−^ or A375 β2m^−^ MR1 cells. Flow cytometry plots of CD25 (x-axis) and CD137 (y-axis) expression on TCR Vδ2-gated cells. Numbers indicate the percentage of positive cells positive in the gated areas. The plots are representative of two independent experiments. **(G)** Flow cytometry plots of CD69 and CD137 expression on T cells from tg mice expressing a human TCR Vγ9Vδ2. Tg mouse T cells were stimulated with A375 β2m^−^, or A375 β2m^−^ MR1 untreated or treated with Zol. Numbers indicate the percentages of cells in each quadrant. Data is representative of four independent experiments. **(H)** Percentage of TCR Vγ9Vδ2 tg mouse cells that upregulated CD69 in the presence of A375 β2m^−^ or A375 β2m^−^ MR1 exposed to Zol or vehicle. Bar plot of mean ± SD of four independent experiments. Each dot represents one experiment and is matched across conditions. Two-way ANOVA, matching measurements, uncorrected Fisher’s LSD test. ^∗∗^ *p*< 0.01; ^∗∗∗^ *p* < 0.001; ns, not significant.

We next studied whether MR1 improves the response of other TCR Vγ9δ2 cells to Zol-treated cells, as observed with the D9A10 clone (**Figure 2J**). We investigated the activation of six TCR Vγ9Vδ2 clones using A375 cells pulsed with Zol in the presence or absence of anti-MR1 mAbs. The addition of anti-MR1 mAbs significantly reduced the amounts of IFN-γ released by the clones D9A10, D1C55, and D1B4 (**Figure 4A**). The effect of anti-MR1 mAbs was negligible for the response of the clones G2B9, D1B5, and D15A3 (**Figure 4A**). The presence of anti-HLA-A, B, and C mAbs, instead, did not have any effect, except for the D1B4 clone (**Figure 4A**). The measurement of antigen-presenting molecule expression on A375 wild-type cells denoted high levels of HLA-A, B, and C molecules, contrasting with the dull expression of MR1 (**Supplementary Figure 4A**), thus showing a strong effect of MR1 despite the low physiological expression.

To further investigate the role of MR1 in facilitating the activation of Vγ9Vδ2 cells, the two additional MR1-enhanced TCR Vγ9Vδ2 cell clones D1C55 and D1B4 were stimulated using A375 β2m^−^ and A375 β2m^−^ MR1 cells, as previously done with the D9A10 clone (**Figure 2J**). Zol-treated APCs expressing MR1 induced an increased response from both clones compared to A375 β2m^−^ cells (**Figure 4B**). Untreated APCs did not stimulate the tested clones. Thus, like D9A10, these T cells are activated more efficiently in the presence of MR1.

### Transfer of TCR Vγ9Vδ2 reconstitutes MR1 responsiveness

To test whether the TCRs Vγ9Vδ2 are involved in the MR1-induced activation, four TCR Vγ9Vδ2 pairs and one MAIT TCR pair were reconstituted in β2m-deficient and TCR-deficient Jurkat (JKT) cells (**Supplementary Figure 4B**). Each transduced JKT cell line was challenged with A375 β2m^−^ cells and A375 β2m^−^ MR1. JKT-D9A10, JKT-D1C55, and JKT-G2B9 showed higher responses to Zol-exposed APCs expressing MR1, while they were non-reactive or showed no different reactivity to vehicle-exposed APC (**Figure 4C**). In contrast, JKT cells expressing the MR1 non-reactive D15A3 TCR did not exhibit differential reactivities to MR1-expressing APCs with or without Zol (**Figure 4D**), confirming the observations with the T cell clones. As an additional control, JKT cells expressing a MAIT TCR (JKT-SMC3), which recognizes MR1-5-OP-RU complexes (45), did not respond to any APCs with or without Zol (**Supplementary Figure 4C**).

The effects of MR1 recognition over Zol-induced activation were also assessed in Zol-titration experiments. The response of JKT-D9A10 cells at high Zol doses to MR1-positive APC was higher than that to MR1-negative APC (**Figure 4E**), directly affecting the efficacy of the response. JKT cells transduced with the non-MR1 reactive TCR D15A3 did not show such an increment (**Figure 4E**). Also, the TCR Vγ9Vδ2 cells derived from the reported patient showed an increased response to MR1-expressing APC. Indeed, up to 36% of TCR Vγ9Vδ2 cells upregulated CD25 and CD137 markers upon challenge with A375 β2m^−^ MR1 cells compared to the 16% background activation observed in co-cultures with MR1-negative A375 cells (**Figure 4F**), confirming the presence of MR1-self-reactive cells.

We further assessed the TCR-dependent recognition of MR1, taking advantage of RAG-2-deficient transgenic (tg) mice expressing the human TCR of the D1C55 clone (21). The tg T cells do not express BTN3-like genes but react in a BTN3A1-dependent manner to pAgs (21). When tg TCR Vγ9Vδ2 cells were stimulated with A375 β2m^−^ cells in the presence of Zol, up to 7.3% of cells upregulated the expression of the activation markers CD69 and CD137 (**Figure 4G**). Upon challenge with A375 β2m^−^ MR1 cells, they showed a marked upregulation of both markers, and the double-positive cells were >30% (**Figure 4G**). In four independent experiments, the response to MR1-expressing APC was significantly higher than that to MR1-negative ones (60% vs. 40%) (**Figure 4H**). Hence, the presence of MR1 was also relevant in this system.

These findings indicated that the TCR Vγ9Vδ2 transfer recapitulates the response to MR1 and confirmed that only some TCRs Vγ9Vδ2 show this capacity. Furthermore, MR1 recognition enhanced the functional response upon TCR engagement.

### MR1 activates TCR Vγ9Vδ2 cells in the absence of BTN3A1

**Figure 5:**
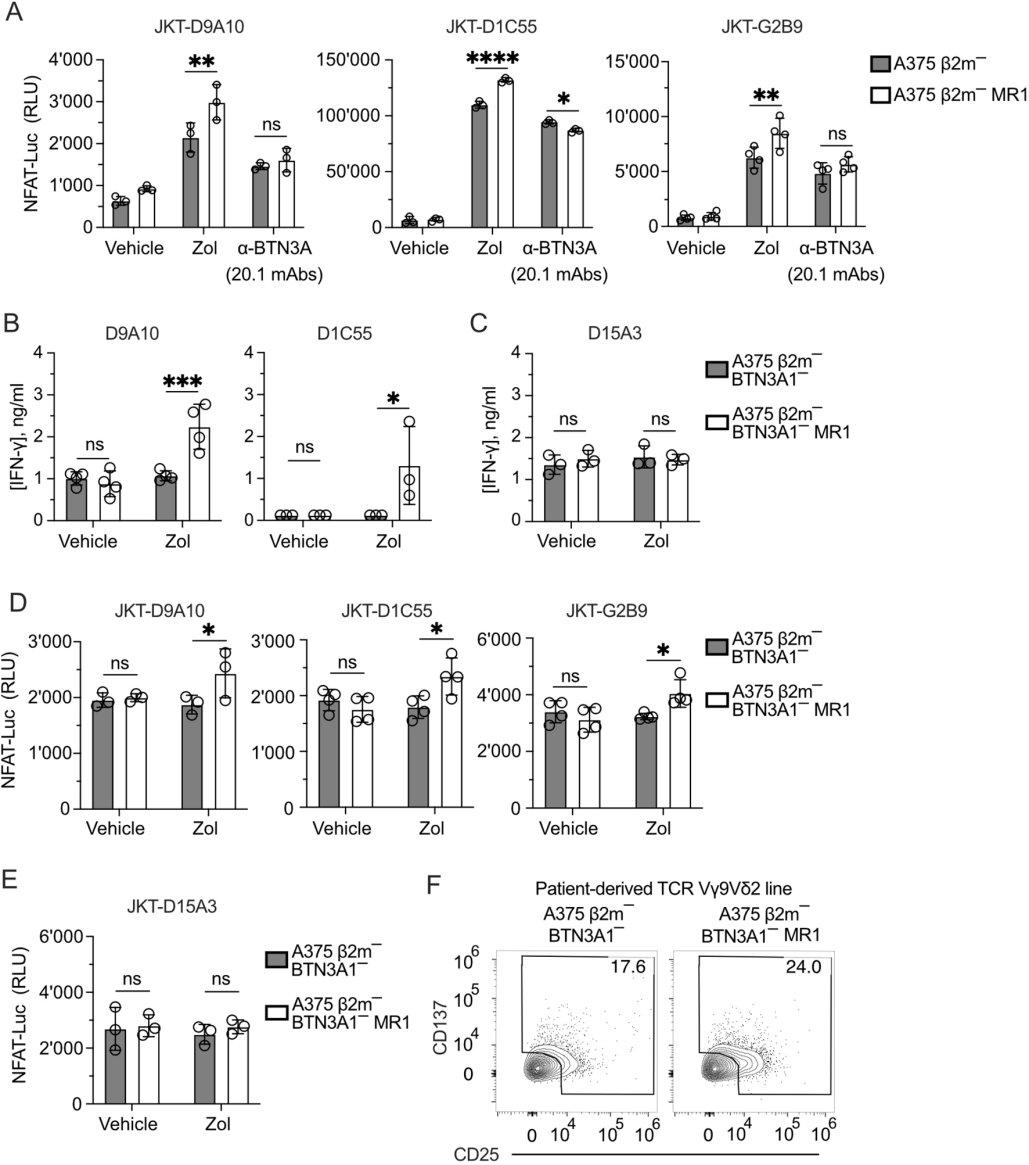
TCRs Vγ9Vδ2 recognize MR1 in the absence of BTN3A1. **(A)** Activation of JKT cells expressing MR1-reactive TCRs Vγ9Vδ2 (D9A10, D1C55, G2B9) challenged with A375 β2m^−^ cells or A375 β2m^−^ MR1 cells exposed to Zol, BTN3A mAbs (clone 20.1) or vehicle. Data is representative of three independent experiments. Plots show the RLU mean ± SD of at least triplicate independent cultures. Two-way ANOVA, Sidak’s multiple comparisons test. ^∗∗^ *p*< 0.01; ^∗∗∗∗^ *p* < 0.0001; ns, not significant. **(B, C)** Response of **(B)** MR1-reactive (D9A10, D1C55) and **(C)** non-MR1-reactive (D15A3) TCR Vγ9Vδ2 clones to A375 β2m^−^/ BTN3A1^−^ cells and A375 β2m^−^/ BTN3A1^−^ MR1 cells exposed to Zol or vehicle. Data is representative of three independent experiments. Plots show the mean IFN-γ (ng/ml) ± SD of at least triplicate independent cultures. Two-way ANOVA, Sidak’s multiple comparisons test. ^∗^ *p* < 0.05; ^∗∗∗^ *p* < 0.001; ns, not significant. **(D, E)** Activation of JKT cells expressing **(D)** MR1-reactive (D9A10, D1C55, G2B9), and **(E)** non-MR1-reactive (D15A3) TCRs challenged with A375 β2m^−^/ BTN3A1^−^ cells and A375 β2m^−^/ BTN3A1^−^ MR1 cells exposed to Zol or vehicle. Data is representative of three independent experiments. Plots show the RLU mean ± SD of at least triplicate independent cultures. Two-way ANOVA, Sidak’s multiple comparisons test. ^∗^ *p* < 0.05; ns, not significant. **(F)** Activation of the patient-derived TCR γδ cell line stimulated with untreated A375 β2m^−^/ BTN3A1^−^ cells or A375 β2m^−^/ BTN3A1^−^ MR1 cells. Flow cytometry plots of CD25 and CD137 expression on TCR Vδ2 gated cells. Numbers indicate the percentage of positive cells. The plots are representative of two independent experiments.

>These results raised two hypotheses: the first is that the Zol-induced pAg accumulation and BTN conformational changes are necessary for the additive effects of MR1 recognition. A second possibility is that Zol treatment induces other cellular changes, which promote the response to MR1 independently of BTNs. Instead of Zol, we performed activation assays with the 20.1 monoclonal antibody, which induces a BTN conformational change and consequent T cell activation (20, 46). This exogenous treatment was performed using A375 β2m− and A375 β2m− MR1 cells to stimulate three JKT cells expressing the three MR1-reacting TCRs D9A10, D1C55, and G2B9 (**Figure 5A**). Under these conditions, there was no increased response to MR1-expressing APCs, thus pointing to Zol-induced changes independent of BTN3A.

We further dissected the role of BTN3A1 by inactivating this gene in A375 β2m^−^ cells (**Supplementary Figures 5A–C**). These cells were then transduced with the MR1-β2m linked gene construct. As expected, the *BTN3A1* gene inactivation abolished the response of D9A10, D1C55 (**Figure 5B**), and D15A3 (**Figure 5C**) clones to Zol-exposed A375 β2m^−^ cells. However, upon Zol exposure, A375 β2m^−^/BTN3A1^−^ cells expressing MR1 activated D9A10 and D1C55 clones (**Figure 5B**). The clone D15A3, which is not sensitive to MR1, did not react to this stimulation (**Figure 5C**). The same experiment was also performed using TCR-transduced JKT cells. JKT cells expressing the D9A10, D1C55, and G2B9 TCRs also responded to Zol-exposed A375 β2m^−^/BTN3A1^−^ cells expressing MR1 (**Figure 5D**), whereas JKT-D15A3 did not react in any of the described conditions (**Figure 5E**).

We then tested the BTN3A1-dependence of the patient’s TCR Vγ9Vδ2 cells and found that when stimulated with BTN3A1-deficient cells, 17.6% of the T cells were activated without MR1 and up to 24% in the presence of MR1 (**Figure 5F**).

In conclusion, these findings showed that MR1 stimulation of the tested TCRs is BTN3A1-independent and suggested that Zol exposure could induce metabolic changes related to the accumulation of MR1-presented antigens.

### MR1-self reactivity of TCR Vγ9Vδ2 cells is Ag-dependent

The effects of Zol could be associated with being an MR1 ligand or with the possibility that it promotes the accumulation of compounds stabilizing MR1. This indirect effect might increase MR1 surface expression, thus activating specific TCR Vγ9Vδ2 cells.

Using a fluorescence polarization-based assay, we disproved the hypothesis that Zol is an MR1-presented Ag. Competition assays were performed with various doses of Zol as a competitor to prevent the binding of JYM20 and a fluorophore-tagged MR1 ligand (47). Zol did not compete even at doses as high as 1 mM. In contrast, Ac-6-FP, a control MR1 ligand, showed efficient competition at 1 µM (**Supplementary Figure 6A**). These findings suggested that Zol does not bind to the JYM20 binding site of MR1.

**Figure 6:**
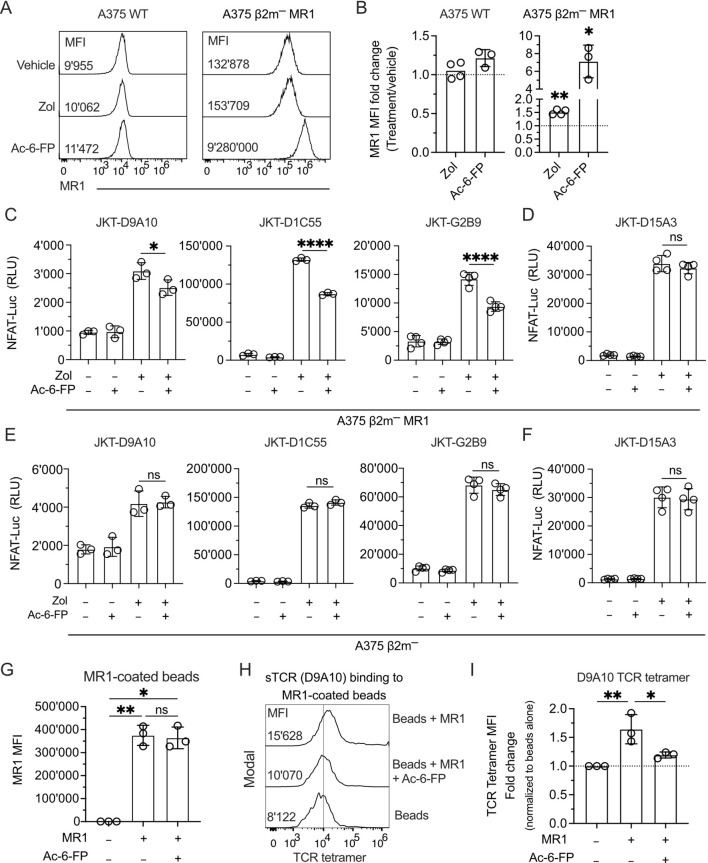
The MR1-presented Ag influences the interaction with the TCR and the activation of TCR Vγ9Vδ2 cells. **(A)** Expression of MR1 on the surface of A375 WT and A375 β2m^−^ MR1 cells measured by flow cytometry after 4 h of pulsing with vehicle, Zol, or Ac-6-FP. The median fluorescence intensity (MFI) of each staining is indicated in the respective histogram. **(B)** Summary of MR1 fold change on A375 WT and A375 β2m^−^ MR1 cells as in **(A)**. MR1 fold change was assessed by normalizing Zol and Ac-6-FP conditions with MFI observed for the vehicle condition. Bar plots illustrate the mean ± SD of at least three independent experiments. Each dot corresponds to one experiment. Two-tailed sample t-test. ^∗^ *p* < 0.05; ^∗∗^ *p*< 0.01. **(C, D)** Activation of JKT cells expressing **(C)** MR1-reactive TCRs (D9A10, D1C55, G2B9) and **(D)** non-MR1-reactive TCRs (D15A3) challenged with A375 β2m^−^ MR1 cells pre-incubated with Ac-6-FP and exposed or not to Zol. Data is representative of three independent experiments. Bar plots illustrate the RLU mean ± SD of at least triplicate cultures. One-way ANOVA, Dunnet’s multiple comparisons test. ^∗^ *p* < 0.05; ^∗∗∗∗^ *p* < 0.0001; ns, not significant. **(E, F)** Activation of JKT cells expressing **(E)** MR1-reactive TCRs (D9A10, D1C55, G2B9) and **(F)** non-MR1-reactive TCRs (D15A3) challenged with A375 β2m^−^ cells pre-incubated with Ac-6-FP and exposed or not to Zol. Data is representative of three independent experiments. Bar plots illustrate the RLU mean ± SD of at least triplicate cultures. One-way ANOVA, Dunnet’s multiple comparisons test. ns, not significant. **(G)** Summary of MR1 expression on the surface of coated and uncoated beads in the presence or absence of Ac-6-FP. Bar plot of MFI mean ± SD of three independent experiments. Each dot corresponds to one experiment. One-way ANOVA, Dunnet’s multiple comparisons test. ^∗^ *p* < 0.05; ^∗∗^ *p*< 0.01; ns, not significant. **(H)** D9A10 TCR tetramer staining of uncoated or MR1-coated beads after pre-treatment or not with Ac-6-FP. Histograms are representative of three independent experiments. MFI is indicated next to each histogram. **(I)** Summary of D9A10 TCR tetramer staining of uncoated or MR1-coated beads in the absence and presence of Ac-6-FP. TCR tetramer MFI was normalized to uncoated beads. Bar plot of mean ± SD of three independent experiments. Each dot corresponds to one experiment. One-way ANOVA, Dunnet’s multiple comparisons test. ^∗^ *p* < 0.05; ^∗∗^ *p*< 0.01.

The second hypothesis was investigated using different approaches. In the first one, A375 WT and A375 β2m^−^ MR1 cells were exposed to Zol for 4 h before evaluating MR1 protein surface levels. Zol treatment induced a 1.5-fold increase in A375 β2m^−^ MR1 compared to untreated cells, whereas MR1 upregulation on A375 WT cells was not statistically significant (**Figures 6A, B**). As a control, Ac-6-FP, a highly potent MR1 binder (48), induced a 1.2-fold MR1 increase on A375 WT and 7-fold on A375 β2m^−^ MR1 cells (**Figures 6A, B**). The MR1 upregulation was unrelated to increased transcription of MR1 since both Zol and Ac-6-FP did not change the relative expression of the *MR1* gene compared to vehicle treatment in A375 WT and A375 β2m^−^ MR1 cells (**Supplementary Figure 6B**). Thus, Zol did not induce increased *MR1* gene transcription and promoted MR1 upregulation through another mechanism.

In the second approach, we assessed if the selected TCRs Vγ9Vδ2 recognize well-known MR1-presented Ags, such as 5-OP-RU and Ac-6-FP (**Supplementary Figure 6C**). Zol-treated A375 β2m^−^ MR1 cells induced the activation of JKT-D9A10, JKT-D1C55, and JKT-G2B9 (**Supplementary Figure 6C**) but not of JKT-SMC3 cells that express a MAIT TCR (**Supplementary Figure 6D**). In contrast, the 5-OP-RU metabolite activated JKT cells expressing the MAIT SMC3 TCR, but none of the TCRs Vγ9Vδ2 (**Supplementary Figure 6D**). Ac-6-FP did not induce a response from any TCR-transduced JKT cells (**Supplementary Figures 6C, D**). We also tested whether MR1 tetramers loaded with different antigens could bind the MR1-reactive TCRs expressed by Jurkat cells. We tested tetramers loaded with Ac-6-FP (48), 5-OP-RU (7), M_1_Ado, M_1_dA, and M_1_Gua (11), which bind MR1 and stimulate specific TCRs.

None of the tetramers stained these cells (**Supplementary Figure 6E**), whereas they efficiently bound Jurkat cells expressing the E8 TCR, which binds MR1 independently of the antigen (36). The same tetramers did not stain the D9A10 and D15A3 clones (**Supplementary Figure 6E**), thus resembling the results observed with JKT cells expressing the same TCRs. These results indicated that the MR1-reactive TCRs Vγ9Vδ2 do not recognize MAIT Ag, Ac-6-FP, or the selected carbonyl adducts, and the tested MAIT TCR does not react to APCs exposed to Zol.

In a third type of experiment, we investigated whether MR1 reactivity is Ag-dependent using Ag competition studies. JKT cells expressing four TCRs were challenged with A375 β2m^−^ MR1 cells incubated with Ac-6-FP before Zol exposure. Ac-6-FP forms stable complexes with MR1 and efficiently competes with the binding of other molecules (48). The Ac-6-FP pre-treatment of A375 β2m^−^ MR1 cells significantly reduced the response of JKT-D9A10, JKT-D1C55, and JKT-G2B9 cells (**Figure 6C**), indicating it was competing with the Ags presented by MR1. Ac-6-FP did not affect the responses of control JKT-D15A3 cells, which are not MR1-self-reactive (**Figure 6D**), and also did not affect the response to Zol-treated MR1-negative cells (**Figures 6E, F**). This data suggests that the MR1-self-reactivity of D9A10, D1C55, and G2B9 TCRs is Ag-dependent.

In a fourth approach, competition experiments were performed using Ac-6-FP, MR1-coated beads, and staining with TCR tetramers. Ac-6-FP treatment did not change the total MR1 on the beads (**Figure 6G**). However, it significantly reduced the binding of the TCR tetramer to MR1 (**Figures 6H, I**).

These findings confirmed that the selected TCR Vγ9Vδ2 binds MR1-Ag complexes, and Ac-6-FP prevented this interaction.

**Figure 7:**
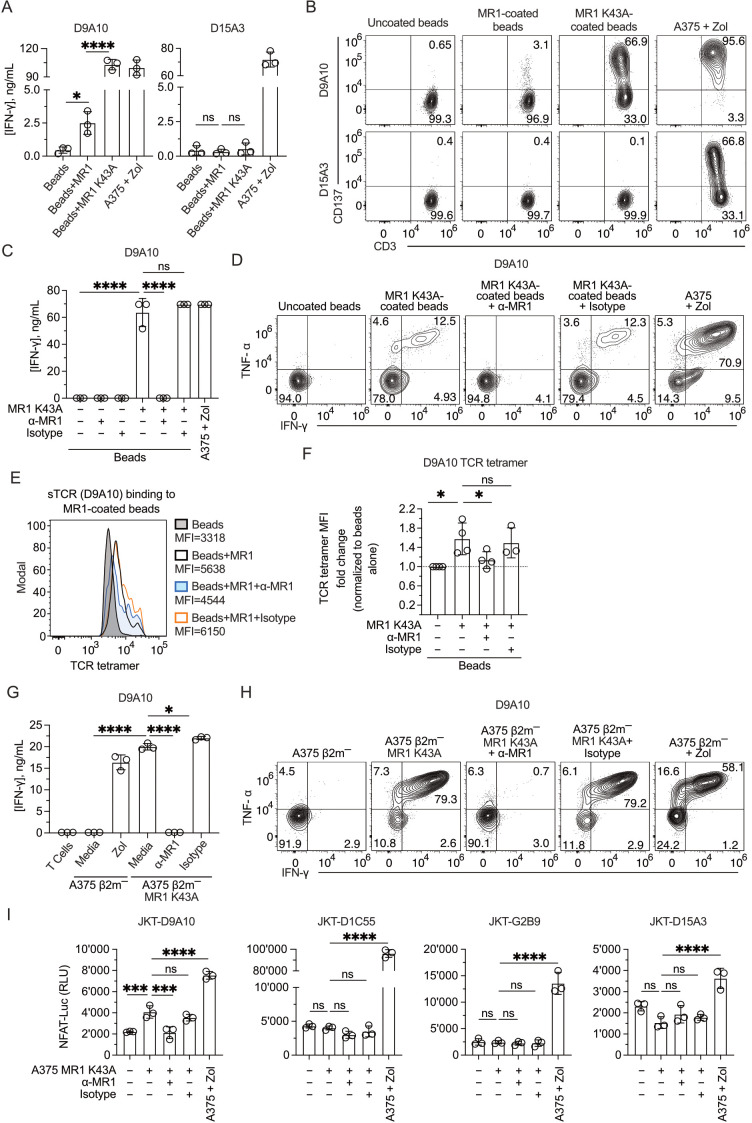
Mutant MR1 K43A stimulates the D9A10 clone better than MR1 WT. **(A)** Activation of D9A10 and D15A3 cell clones using beads coated or not with soluble MR1 WT or K43A mutant. Both clones were stimulated with A375 cells treated with Zol (10µM) as a positive control. Data is representative of three independent experiments. Bar plots of IFN-γ release mean ± SD of triplicate independent cultures. One-way ANOVA, Dunnett’s multiple comparisons test. ^∗^ *p*< 0.05; ^∗∗∗∗^ *p*< 0.0001; ns, not significant. **(B)** Activation of D9A10 upon challenge with MR1 WT-, MR1 K43A-coated or uncoated beads. As a positive control, the clone was challenged using A375 cells treated with Zol (10µM). Flow cytometry plots show the expression of CD3 (x-axis) and CD137 (y-axis). Numbers indicate percentages of cells in the quadrants. Data is representative of three independent experiments. **(C)** Activation of D9A10 clone using beads coated or not with soluble MR1 K43A, in the presence or absence of anti-MR1 or isotype-matched mAbs. As a positive control, the clone was challenged with A375 cells treated with Zol (10µM). Data is representative of three independent experiments. Bar plots of IFN-γ release mean ± SD of triplicate independent cultures. One-way ANOVA, Dunnett’s multiple comparisons test. ^∗∗∗∗^ *p*< 0.0001; ns, not significant. **(D)** Intracellular staining of IFN-γ and TNF-α in D9A10 clone after stimulation using beads coated or not with soluble MR1 K43A, in the presence or absence of anti-MR1 or isotype-matched mAbs. As a positive control, the clone was challenged with A375 cells treated with Zol (10µM). Data is representative of three independent experiments. **(E)** Staining of MR1 K43A-coated or uncoated beads using D9A10 TCR tetramer in the presence or absence of anti-MR1 or isotype-matched mAbs. Histograms are representative of at least three independent experiments. Median fluorescence intensity (MFI) is indicated next to each histogram. **(F)** Summary of D9A10 TCR tetramer staining of MR1 K43A-coated or uncoated beads in the absence and presence of anti-MR1 or isotype-matched mAbs. TCR tetramer MFI was normalized to uncoated beads. Bar plot of mean ± SD of at least three independent experiments. Each dot represents an independent experiment. One-way ANOVA, Dunnet’s multiple comparisons test. ^∗^ *p*< 0.05; ns, not significant. **(G)** Activation of the D9A10 clone challenged with A375 β2m^−^ cells and A375 β2m^−^ MR1 K43A cells in the presence or absence of anti-MR1 or isotype-matched mAbs. As a positive control, the clone was challenged with A375 cells treated with Zol (10µM). Data is representative of three independent experiments. Bar plots of IFN-γ release mean ± SD of triplicate independent cultures. One-way ANOVA, Dunnett’s multiple comparisons test. ^∗^ *p*< 0.05; ^∗∗∗∗^ *p*< 0.0001. **(H)** Intracellular staining of IFN-γ and TNF-α in D9A10 clone challenged with A375 β2m^−^ cells and A375 β2m^−^ MR1 K43A cells, in the presence or absence of anti-MR1 or matched isotype mAbs. As a positive control, the clone was challenged using A375 cells treated with Zol (10µM). Numbers indicate percentages of cells in the quadrants. Data is representative of three independent experiments. **(I)** Activation of JKT cells expressing MR1-reactive TCRs (D9A10, D1C55, G2B9) and non-MR1-reactive TCRs (D15A3) challenged with A375 β2m^−^ cells and A375 β2m^−^ MR1 K43A cells, in the presence or absence of anti-MR1 or isotype-matched mAbs. As a positive control, the clone was challenged with A375 cells treated with Zol (10µM). Data is representative of three independent experiments. Bar plots illustrate the RLU mean ± SD of triplicate cultures. One-way ANOVA, Dunnet’s multiple comparisons test. ^∗∗∗^ *p* < 0.001; ^∗∗∗∗^ *p* < 0.0001; ns, not significant.

In a fifth approach, we tested T cell reactivity to MR1 mutated at lysine 43, located within the antigen-binding pocket of MR1 (7). Beads coated with MR1 WT or the MR1 K43A mutant were compared in activation experiments of the D9A10 and D15A3 clones. Surprisingly, the beads coated with MR1 K43A stimulated D9A10 cells very efficiently, as detected by IFN-γ release (**Figure 7A**) and CD137 marker upregulation (**Figure 7B**). In contrast, the MR1 non-reactive D15A3 cells were not stimulated (**Figures 7A, B**). Control experiments showed that MR1 WT and K43A were equally bound to the beads (**Supplementary Figure 7A**).

The bead-induced activation of D9A10 cells was entirely blocked by the addition of anti-MR1 antibodies, not by isotype-matched antibodies, in the IFN-γ release assay (**Figure 7C**) and intracellular staining for IFN-γ and TNF-α (**Figure 7D**). The same assays showed no stimulation by MR1 K43A on the D15A3 clone (**Supplementary Figures 7B C**).In further experiments, beads coated with MR1 K43A were stained with TCR tetramers, and this interaction was blocked by anti-MR1 antibodies, not by isotype-matched ones (**Figures 7E, F**), thus further validating MR1 reactivity with the D9A10 TCR.

The stimulatory activity was next investigated using A375 β2-m-deficient cells expressing the MR1 K43A mutant (**Supplementary Figure 7D**). These APCs were potent stimulators of D9A10 cells, and the anti-MR1 antibodies, not isotype-matched antibodies, blocked IFN-γ release (**Figure 7G**) and intracellular accumulation of IFN-γ and TNF-α (**Figure 7H**). The control D15A3 clone did not respond in these assays (**Supplementary Figures 7E, F**).

Finally, we tested whether JKT cells expressing the MR1-reactive TCRs D9A10, D1C55, and G2B9 also showed a response to MR1 K43A. The JKT-D9A10 confirmed the response to this MR1 mutant, which was blocked by adding anti-MR1 and not isotype-matched antibodies (**Figure 7I**). In contrast, the other two MR1-reactive TCR cell lines and the control D15A3 did not respond (**Figure 7I**).

Together, these experiments indicate that the antigens presented by MR1 are necessary for activating select TCR Vγ9Vδ2. Some antigens do not require the presence of lysine 43 in the antigen-binding pocket, resembling what was observed with some MR1 T cell clones (9).

### CDR3δ participates in MR1-reactivity

**Figure 8:**
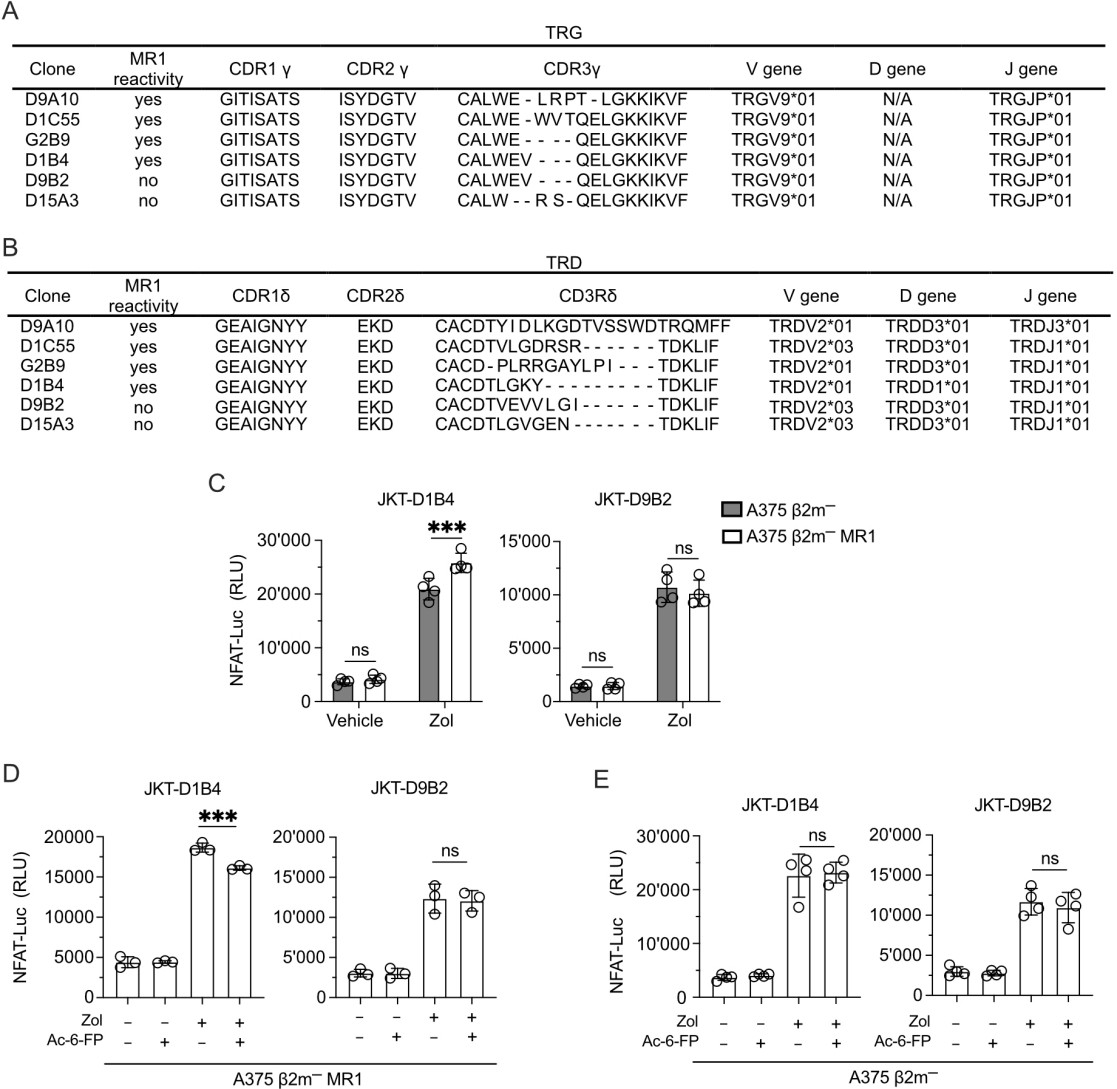
The Vδ2 CDR3 is relevant for MR1 recognition. **(A, B)** Gene usage and amino acid sequences of the **(A)** CDR γ and **(B)** CDR δ of MR1-reactive and non-MR1-reactive TCR Vγ9Vδ2 cell clones. **(C)** Activation of JKT cells expressing MR1-reactive (D1B4) and non-MR1-reactive (D9B2) TCRs Vγ9Vδ2 challenged with A375 β2m^−^ cells (black bars) or A375 β2m^−^ MR1 cells (white bars) exposed to Zol or vehicle. Data is representative of three independent experiments. Plots show the RLU mean ± SD of quadruplicate independent cultures. Two-way ANOVA, Sidak’s multiple comparisons test. ^∗^ *p*< 0.05; ns, not significant. **(D, E)** Activation of JKT cells expressing MR1-reactive (D1B4) and non-MR1-reactive (D9B2) TCRs challenged with **(D)** A375 β2m^−^ MR1 cells, or **(E)** A375 β2m^−^ cells pre-incubated with Ac-6-FP and exposed or not to Zol. Data is representative of three independent experiments. Bar plots illustrate the RLU mean ± SD of at least triplicate cultures. One-way ANOVA, Dunnet’s multiple comparisons test. ^∗∗^ *p* < 0.01; ^∗∗∗^ *p* < 0.001; ns, not significant.

TCR sequencing of the clones investigated in the study all showed the use of TRGV9*01-TRGJP*01 rearrangements and different CDR3 regions (**Figure 8A**). Instead, they showed TRDV2 genes rearranged to TRDJ3*01 or TRDJ1*01 and different CDR3 regions (**Figure 8B**). The clones D1B4 and D9B2 shared an identical TCR Vγ9 chain but expressed TCR Vδ2 chains with different CDR3 regions (**Figures 8A, B**). Only the D1B4 clone was MR1-self-reactive, raising the possibility that MR1 reactivity depended on CDR3δ. The reconstitution of both TCRs in JKT cells (**Supplementary Figure 8A**) confirmed the lack of reactivity of the D9B2 Vδ2 chain (**Figure 8C**). Due to the involvement of the TCR CDR3 in Ag interactions, we tested the Ag relevance of this recognition. Competition experiments with Ac-6-FP reduced the recognition of Zol-treated A375 β2m− MR1 APCs (**Figure 8D**). Instead, in control experiments, the recognition of A375 β2m− APC was not inhibited (**Figure 8E**). This data suggests that the TCR Vδ2 chain participates in MR1 reactivity and that its CDR3 region plays a relevant role.

## Discussion

A partially resolved immunological question is how T cells expressing TCRs γδ are physiologically activated. Recent studies highlighted that the Vγ4 or Vγ9 chains interact with different members of the butyrophilin family and induce T cell activation resembling those of innate-like receptors (33, 34). Other studies reported rare single TCR γδ cell clones recognizing classical MHC, CD1, and MR1 molecules, reviewed in (12, 14, 34, 49, 50). This rare occurrence keeps open the question of whether the majority of TCRs γδ are activated by innate-like mechanisms or, instead, some of these cells also recognize complexes formed by Ag-presenting molecules and individual Ags, like TCRs αβ (51). Lack of extensive evidence for broad “classical” Ag recognition, together with the possibility of assembling long CDR3δ regions resembling those of immunoglobulins and much less those of TCR αβ, suggested that the TCR γδ is selected for the recognition of nonlinear epitopes featuring the Ag recognition mode of immunoglobulins (51).

In some cases, the mode of assembly and Ag recognition by TCR γδ is unique. One study reported an example of a TCR γδ that forms super dimers after the interaction between two Vγ5 chains (52). This TCR dimerization was necessary for TCR signaling. Whether this unusual TCR assembly on the cell surface applies to other TCRs γδ remains to be investigated. It might explain the difficulty in identifying target molecules using soluble TCRs γδ if it occurs frequently. The same study showed that a TCR Vγ9Vδ2 does not form super dimers and is characterized by high flexibility in the V-J regions. Such flexibility was also reported in the V-J region of a TCR Vγ8Vδ3 (53). In the case of the TCR Vγ9Vδ2, this plasticity is necessary for the interaction with BTNs.

Within T cells expressing the TCR Vγ9Vδ2, there is even smaller evidence that their Ag recognition mimics that of adaptive TCR αβ cells (54–56). The most effective TCR Vγ9Vδ2-mediated cell activation relies on TCR interaction with BTN3A1, BTN3A2, and BTN2A1 molecules expressed by target cells. This type of stimulation is considered innate-like (33), as the CDR3 region of the Vγ9 chain is not relevant (27, 29, 31). Indeed, BTN2A1 interacts with a TCR Vγ9 chain framework region (23, 24). The Vδ2 chain instead interacts with BTN3A2 in complex with BTN3A1 (32).

The CDR3 regions of the Vδ2 chain are the most variable among B and T cell receptors. Indeed, they can include in-frame rearrangement of all TRDD regions, originating highly polyclonal TCRs Vγ9Vδ2 as a consequence of somatic recombination. The innate-like structural and functional features of the TCR Vγ9Vδ2 interaction with BTNs raise the question of what forced the TCRs Vδ2 chains in these T cells to maintain such CDR3 diversity. One possibility is that these CDRs may interact with complexes formed by antigen-presenting molecules and Ags that remain unknown because they appear only in some cell types or during cellular stress.

Our studies reveal that some TCRs Vγ9Vδ2 mediate T cell activation with a mechanism similar to adaptive TCRs. The importance of the presented Ag and the CDR3δ supports this finding. The cognate interaction between TCR and MR1 was confirmed with soluble TCR and tumor-cell-derived soluble MR1 molecules. As detected by mass spectrometry studies, the secreted MR1 molecules present different Ags (11, 57), including small metabolites generated in the nucleoside and carbonyl pathways (11). This heterogeneity prevented us from measuring the affinity of the interaction with the soluble TCR γδ. The relevance of this direct binding was confirmed by MR1-reactive TCR gene transfer that reconstituted MR1 recognition. Thus, MR1-Ag complexes are additional stimulators of TCR γδ cells in an Ag-dependent manner. The MR1-reacting cells are not confined to rare individual clones but are a small cell population detectable *ex vivo* with soluble MR1. The interaction between select TCRs Vγ9Vδ2 and MR1 differs from those reported for other TCRs γδ interacting with MR1 for which the Ag does not have a detectable role (12, 14).

One critical issue is the nature of the MR1-presented Ags that stimulate TCR Vγ9Vδ2 cells. MR1 can accommodate a variety of Ags with highly divergent structural features (6, 7, 11, 57, 58). Thus, it is challenging to anticipate which family of molecules might be stimulatory. The function of TCR Vγ9Vδ2 cells has been associated with the response to cells that, under stress, change the expression of surface molecules (59). This possibility might also apply to recognizing MR1-presented carbonyl adducts of nucleobases, accumulating in cells with simultaneously altered nucleotide and carbonyl metabolic pathways (11). In line with this possibility is the observation that Zol exposure promotes the response to MR1 independently from BTN3A1. In addition to inhibiting the mevalonate pathway, this drug has other effects, including the induction of oxidative stress, ROS production, and glutathione depletion (60–62). These metabolic changes contribute to the accumulation of the carbonyl nucleoside adducts presented by MR1 (11). Therefore, Zol might promote the generation of MR1-binding Ags, some of which activate MR1-restricted TCR Vγ9Vδ2 cells. This mechanism aligns with the observed increase in surface expression of the MR1 protein following incubation with Zol and the observation that the additive effect of MR1 is not observed when BTN agonist antibodies are used as activating reagents.

**Figure 9:**
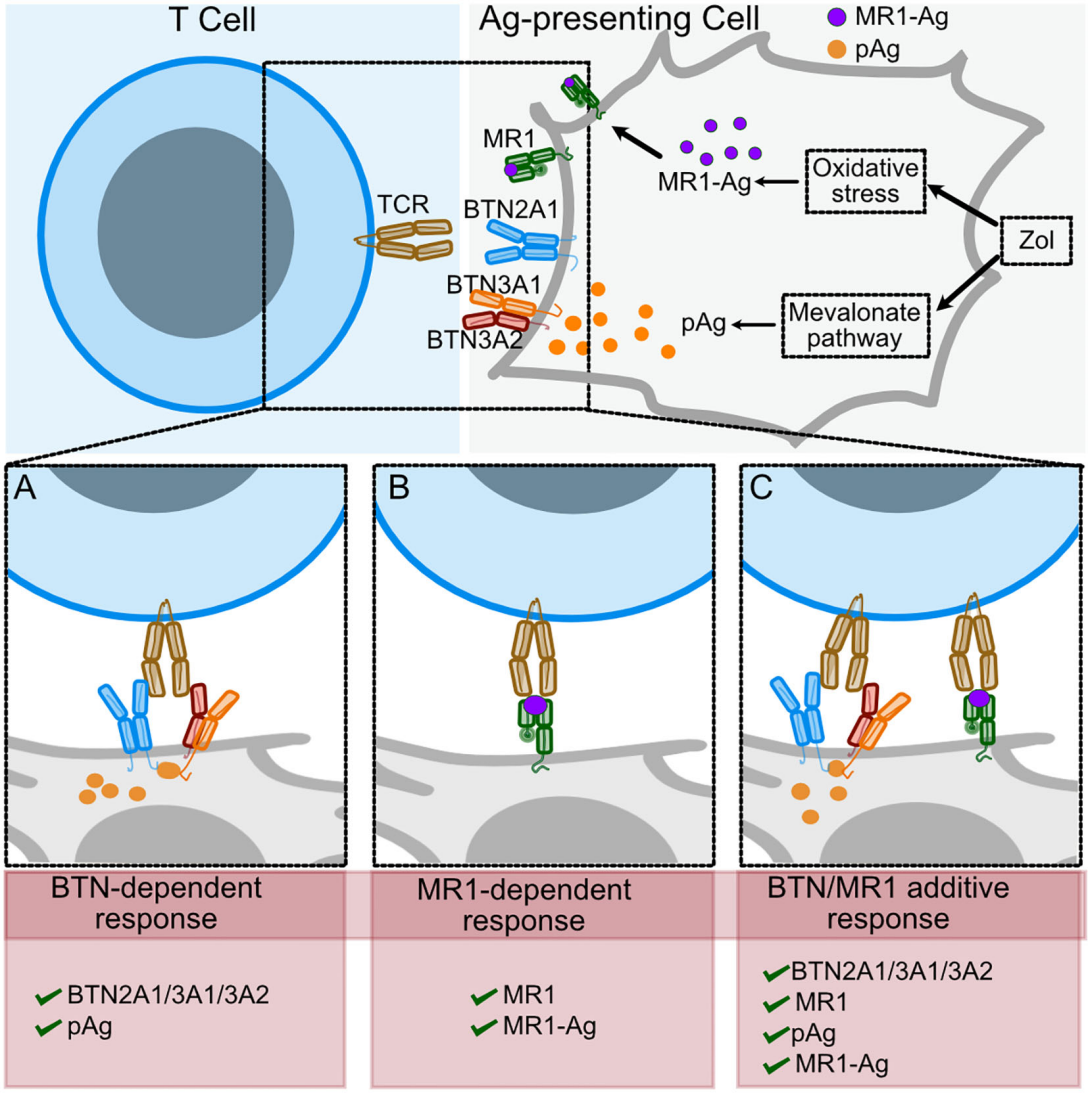
Scheme of TCR Vγ9Vδ2 recognition of BTNs in the presence of pAgs or of MR1-Ag complexes. Proposed model of Zol effects on T cell activation. Zol exposure induces changes in the mevalonate pathway that lead to pAg accumulation and other cellular changes, including oxidative stress (upper panel). **(A)** In the presence of pAg, the TCR interacts with BTN2A1 homodimers and BTN3A1/3A2 heterodimers. **(B)** Some TCR Vγ9Vδ2 interact with MR1-Ag complexes. **(C)** When both pAg and stimulating MR1-Ag complexes engage the TCRs on the same cell, the T cell response increases.

The relevance of the Ag is also indicated by the efficient stimulation of the D9A10 cells by the MR1 K43A mutant. This MR1 is mutated within the antigen-binding pocket and binds antigens that do not form a Schiff’s base (7, 58). The fact that it efficiently stimulates D9A10 cells, not other MR1-reactive TCR Vγ9Vδ2 cells, further suggests that individual TCRs recognize different antigens. In conclusion, we propose that Zol has two mechanisms of action. It induces the accumulation of pAgs, which promote T cell activation through conformational changes of BTNs. In addition, Zol exposure promotes metabolic alterations, increasing the availability of antigens presented by MR1 (**Figure 9**).

A second issue is the frequency of MR1-restricted TCR Vγ9Vδ2 cells. Based on the activation assay using MR1-coated beads, we estimated frequencies between 0.08 and 1.64% among circulating TCR Vγ9Vδ2 cells in healthy donors. These frequencies are merely indicative because they reveal T cells activated in non-optimal conditions due to a lack of adequate co-stimulation and the probable absence of potent Ags stimulating the TCRs in the sample. Nevertheless, this reactivity remains broader than that observed with peptide-specific TCR αβ cells (63, 64).

Our data also indicates that MR1-restricted TCR Vγ9Vδ2 cells may expand under specific conditions. In one patient with TCR γδ lymphocytosis, 10.7% of circulating T cells were MR1-autoreactive TCR Vγ9Vδ2-positive. These cells expressed activation markers *ex vivo*, indicating previous Ag experience, and showed a functional phenotype skewed towards producing type I cytokines. Further studies and additional patients must be investigated to assess whether these cells are clinically relevant.

In addition to the potential role in disease, the physiological role of MR1-restricted TCR γδ cells remains to be defined. By recognizing self-antigens, these cells might contribute to tissue integrity and surveillance, promoting tissue repair and immune protection from bacterial infections (65).

A final important aspect is that the MR1-autoreactive TCR Vγ9Vδ2 cells also interact with BTNs. Thus, these cells can be activated by interaction with BTNs in the presence of pAgs or by specific MR1-Ag complexes. Both mechanisms can be present in some circumstances, resulting in enhanced effector functions (**Figure 9**). This dual mode of concurrent stimulation might have opposite effects. On one hand, it might provide the advantage of increased responses during bacterial infection or tumor cell recognition. On the other hand, it may have the disadvantage of increasing the risk of autoimmune responses. Future studies involving more patients may address this issue adequately.

## Data Availability

The original contributions presented in the study are included in the article/**Supplementary Material**. Further inquiries can be directed to the corresponding author.
