# Supplementary material for: Recognition of MR1-antigen complexes by TCR Vγ9Vδ2
Source: Front Immunol. 2025 Feb 18;16:1519128. doi: 10.3389/fimmu.2025.1519128 (PMC11876030; doi:10.3389/fimmu.2025.1519128)
Supplement: Supplementary file 1 [file DataSheet1.pdf]

Supporting Information

Recognition of MR1-antigen complexes by TCR Vγ9Vδ2

José Pedro Loureiro[1], Alessandro Vacchini[1], Giuliano Berloffa[1], Jan Devan[1], Verena Schaefer[1], Vladimir Nosi[1], Rodrigo Colombo[1], Aisha Beshirova[1], Giulia Montanelli[1], Benedikt Meyer[2], Timothy Sharpe[3], Andrew Chancellor[1], Mike Recher[2], Lucia Mori[1], Gennaro De Libero[1]*

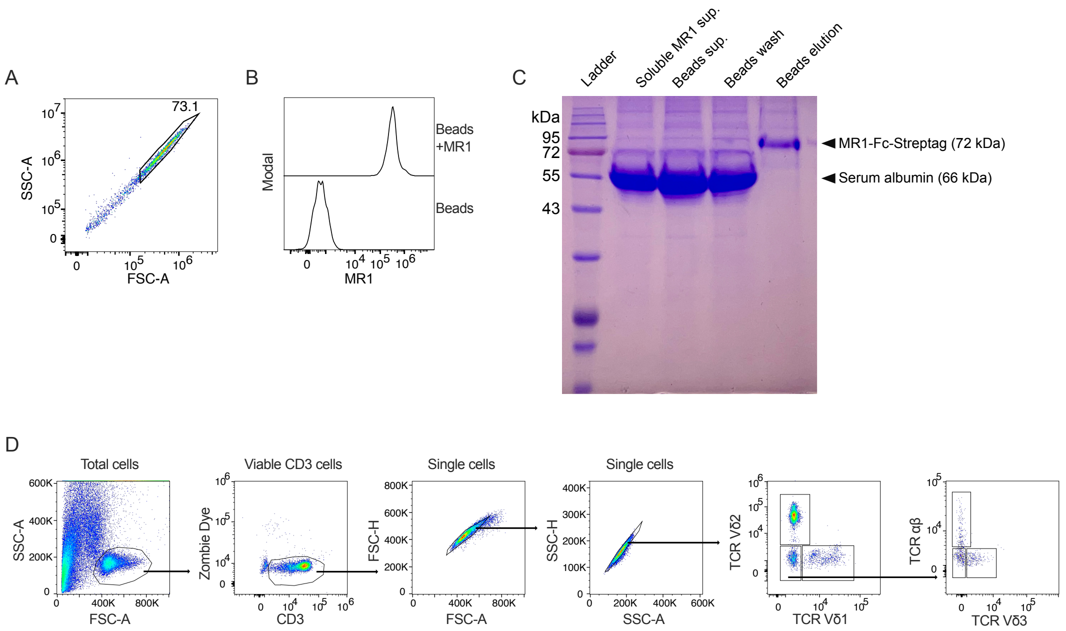

Supplementary Figure 1.

Quality control of MR1-coated beads and gating strategy for PBMC-isolated TCR γδ cells.

(A) Flow cytometry gating strategy of MR1-coated beads based on SSC-A and FSC-A.

(B) Histograms of MR1 expression on MR1-coated beads (Beads + MR1) and uncoated beds (Beads) using the anti-MR1 (26.5) mAbs.

(C) SDS-PAGE analysis of the loaded and eluted samples from streptactin-coated magnetic beads. Lane 1 – molecular weight marker; lane 2 – supernatant of A375 producing soluble MR1; lane 3 – supernatant collected from beads after 18 h incubation; lane 4 – material eluted after washing beads using PBS; lane 5 – material eluted from the streptactin-coated magnetic beads using biotin (100 µM). Samples 2, 3 and 4 contain predominantly albumin (66 kDa), whereas sample 5 mainly comprises a protein with a mass consistent with the monomeric β2m-MR1 Fc-Streptag (72 kDa).

(D) Gating strategy of negatively-enriched TCR γδ cells isolated from PBMCs of healthy donors. Lymphocytes were sequentially gated according to FSC-A/SSC-A, and viable CD3 cells ($CD3^+$ / Live dead excluded with Zombie dye). Singlets were selected using FSC-A/ FSC-H and SSC-A/SSC-H, and the expression of TCR Vδ1, Vδ2, Vδ3, and TCR αβ was determined on the cells resulting from the sequential gates.

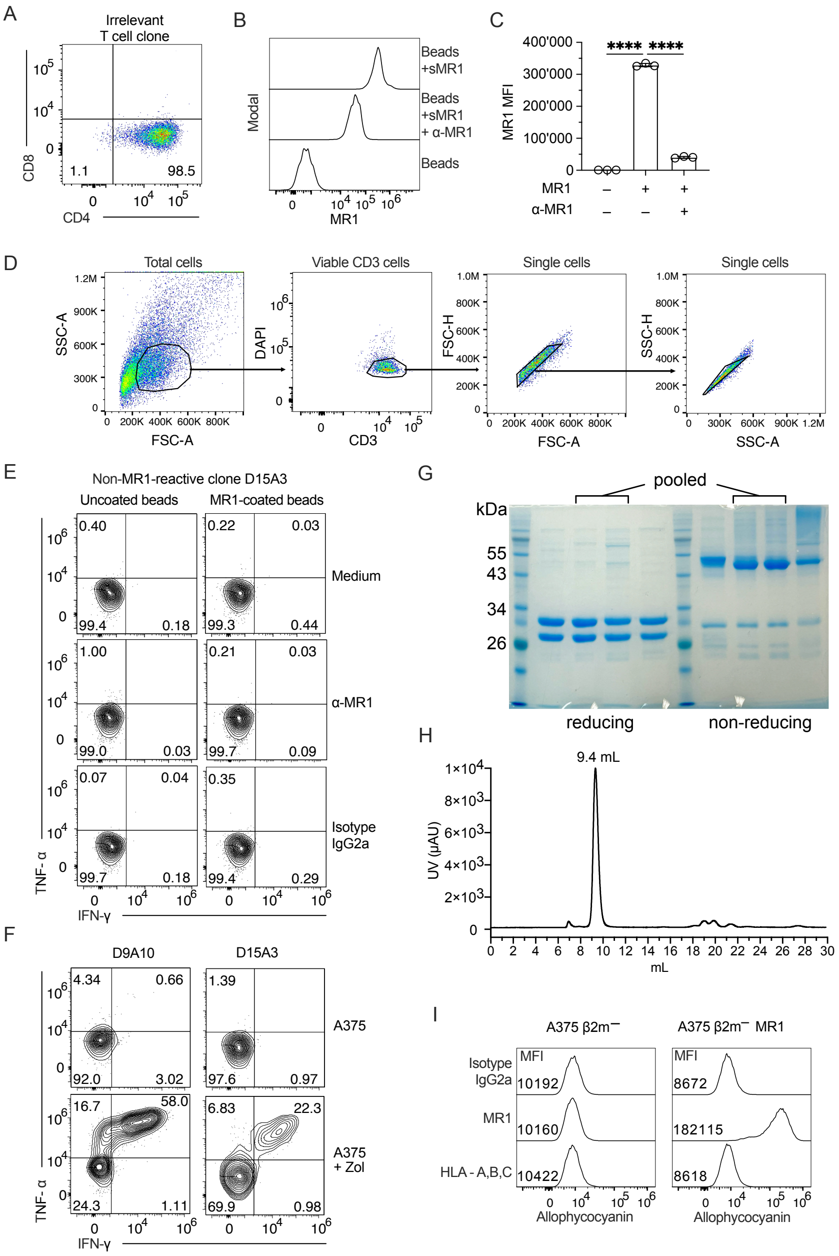

Supplementary Figure 2.

T cell clone characterization, MR1 blocking on MR1-coated beads, D15A3 activation, and purity of TCR D9A10 monomer.

(A) CD4 and CD8 expression on an irrelevant TCR αβ cell clone used as a negative control.

(B) Expression of MR1 on uncoated beads (Beads) and MR1-coated beads treated or not with anti-MR1 (26.5) mAbs.

(C) Summary of MR1 expression on the surface of MR1-coated and uncoated beads incubated or not with anti-MR1 mAbs. MFI, median fluorescence intensity. Bar plot of mean ± SD of three independent experiments. One-way ANOVA, Dunnet's multiple comparisons test. $^{****}p < 0.0001$.

(D) Gating strategy of D9A10 clone activated with beads under different conditions. Cells were gated according to FSC-A/SSC-A, then on viable CD3 cells ($CD3^+$ DAPI). Singlets were selected on FSC-A/ FSC-H and SSC-A/SSC-H.

(E), (F) Intracellular staining of IFN-γ and TNF-α in **(E)** D15A3 clone after stimulation using beads coated or not with soluble MR1, in the presence or absence of anti-MR1 or isotype-matched mAbs or in **(F)** D9A10 and D15A3 clones after stimulation with A375 treated or not with Zol (10µM). Data in E and F are representative of three independent experiments.

(G) SDS-PAGE analysis of the eluted fractions from ion exchange chromatography purification of D9A10 soluble TCR, under reducing and non-reducing conditions.

(H) Size-exclusion chromatography analysis of biotinylated soluble D9A10 TCR.

(I) MR1 and HLA A, B, C expression on A375 $\beta 2m^-$ and A375 $\beta 2m^-$ MR1 cells by flow cytometry. MR1, HLA A, B, C, and isotype-matched irrelevant mAbs staining are shown, and their median fluorescence intensity (MFI) is indicated next to each histogram. Staining was repeated when cells were kept in culture for two weeks to confirm stable MR1 expression.

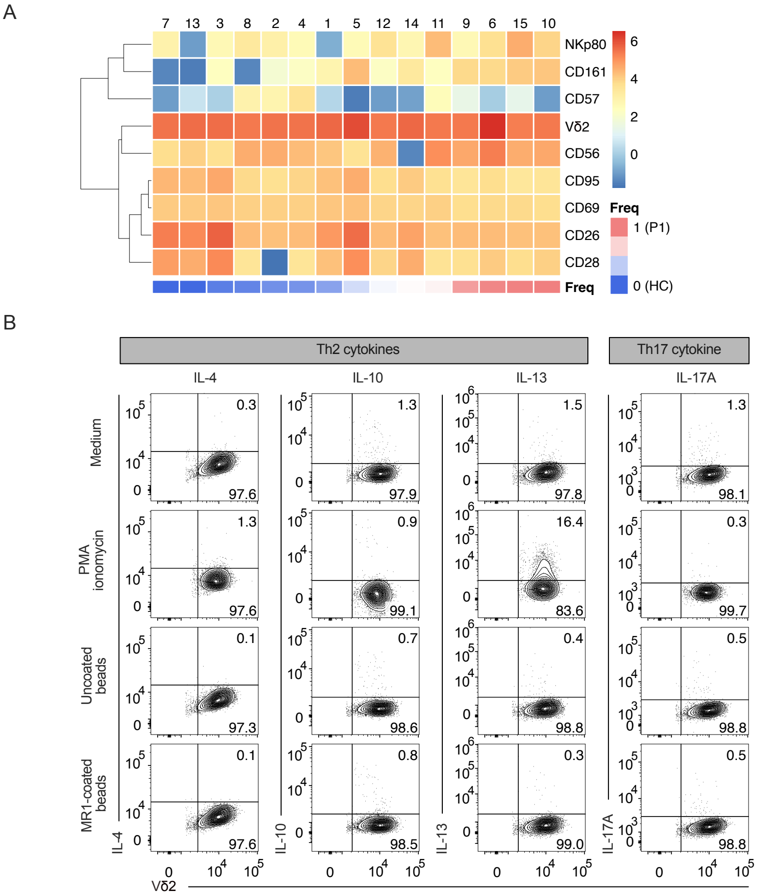

Supplementary Figure 3.

Phenotypic and functional characterization of patient-derived TCR Vδ2 cells.

(A) Heatmap of UMAP clustering characterization of TCR Vδ2 cells from two HCs and the patient with TCR γδ cell lymphocytosis, distributed in clusters 1 to 15 according to the expression of CD161, CD26, CD69, CD95, CD28, CD57, NKp80 and CD56 markers. Color scales on the right indicate the relative expression of the markers and frequency in patient P1 and healthy controls HC.

(B) Activation of a patient-derived TCR γδ cell line stimulated or not with PMA/ionomycin, uncoated or MR1-coated beads. Flow cytometry plots of intracellular IL-4, IL-10, IL-13 (Th2 cytokines), IL-17A (Th17 cytokine) on the y-axis and TCR Vδ2 (x-axis).

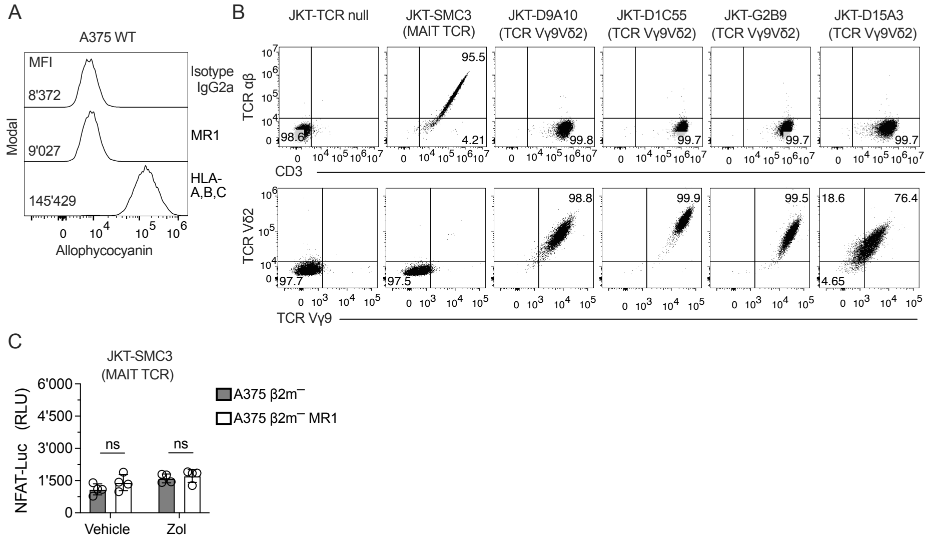

Supplementary Figure 4.

Expression of MR1 molecules on the surface of A375 cells and TCRs Vγ9Vδ2 on transduced JKT cells.

(A) MR1 and HLA A, B, C expression on A375 WT cells by flow cytometry. The allophycocyanin median fluorescence intensity (MFI) of each staining is indicated on the left of each histogram.

(B) Expression of one MAIT (SMC3) and four Vγ9Vδ2 (D9A10, D1C55, G2B9, D15A3) TCRs on JKT cells. Flow cytometry plots of TCR αβ and CD3 (top plots) and Vδ2 and Vγ9 (bottom plots).

(C) Activation of JKT-SMC3 cells (expressing a MAIT cell TCR) challenged with A375 β2m⁻ cells (black columns) or A375 β2m⁻ MR1 cells (white columns) exposed to Zol or vehicle. Data is representative of three independent experiments. Plots show the RLU mean ± SD of quadruplicate independent cultures. Two-way ANOVA, Sidak's multiple comparisons test. ns, not significant.

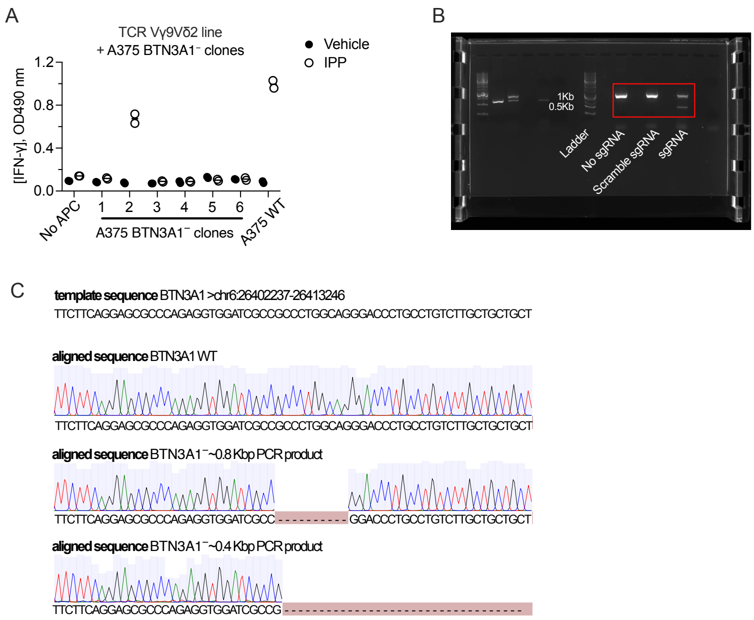

Supplementary Figure 5.

Generation of BTN3A1-deficient A375 cells.

(A) Screening of BTN3A1-deficient A375 cell clones. Activation of a TCR Vγ9Vδ2 cell line challenged with A375 cell clones in the presence or absence of IPP. Scatter plots of IFN-γ release. Each dot represents a duplicate culture performed using individual clones of BTN3A1-inactivated A375 cells.

(B) Agarose gel electrophoresis of PCR product of different A375 cells flanking the target region of the used sgRNA. Lane 1 - Ladder with size marker; Lane 2 - A375 cells untreated with sgRNA; Lane 3 - A375 cells treated with Scramble sgRNA; Lane 4 - A375 cells treated with sgRNA targeting BTN3A1. The gel is uncropped, and the lanes on the ladder's left side are unrelated to this experiment.

(C) Alignment of the flanked BTN3A1 gene that is targeted by the sgRNA with sequence available on GenBank (Gene ID: 11119) by using Benchling [Biology Software] (2024). The flanked sequences of A375 WT cells and the 0.8 Kbp and 0.4 Kbp sequences obtained from the sgRNA-treated A375 BTN3A1- cell clone 6 are shown aligned with the sequence in the public data set (top row).

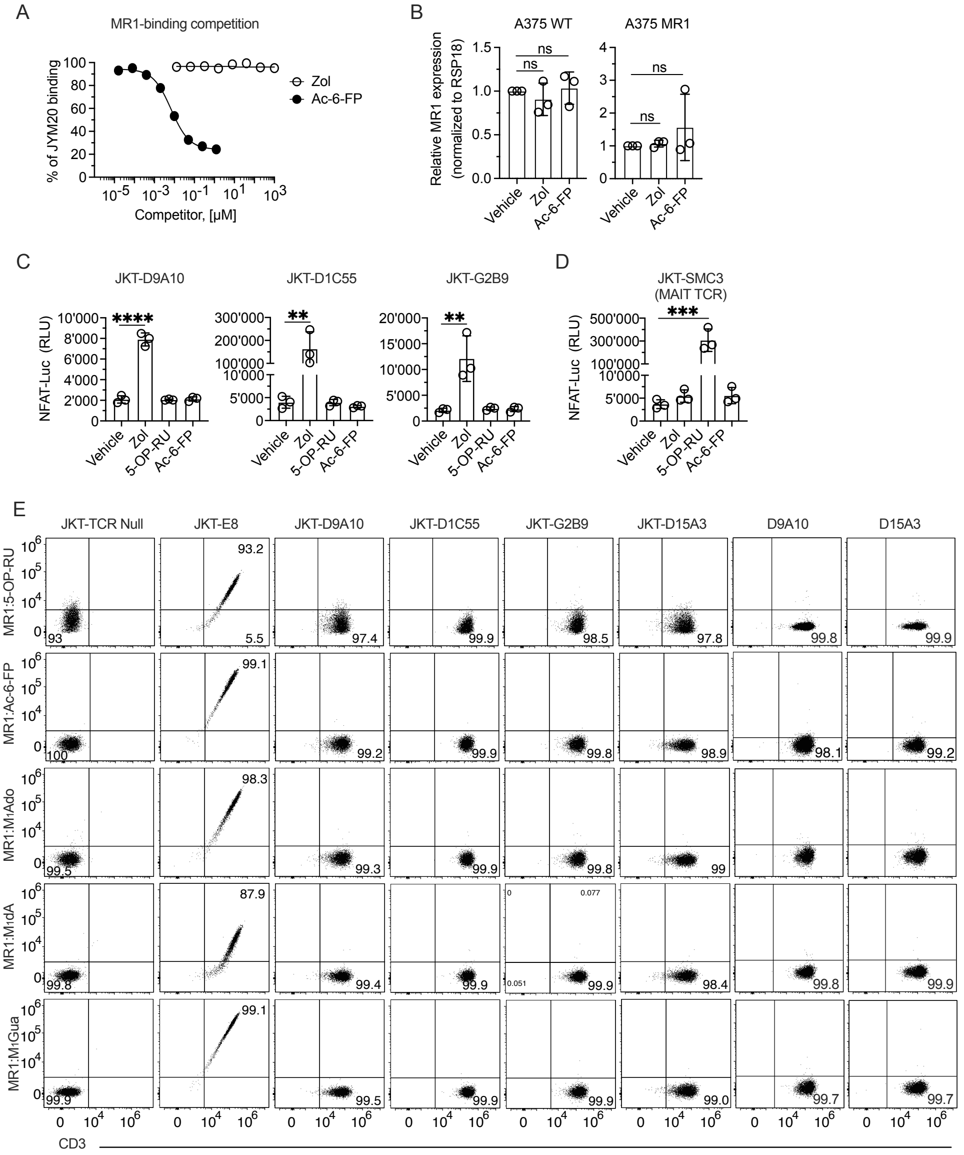

Supplementary Figure 6.

ZOL does not compete with other MR1 binders, and 5-OP-RU nor Ac-6-FP stimulate JKT cells expressing MR1-reactive TCRs Vγ9Vδ2 or bind MR1-Ag tetramers.

(A) Competition assay using Zol (open circles) or Ac-6-FP (closed circles) and the MR1-fluorescent ligand JYM20. Each symbol represents the mean of duplicate measurements observed in two independent experiments. The y-axis shows the percentage of the JYM20 fluorescence observed relative to JYM20 alone.

(B) Relative MR1 gene expression in A375 WT and A375 β2m⁻ MR1 cells incubated with Zol, Ac-6-FP or vehicle. Gene expression was calculated as $2^{(-\Delta\Delta Ct)}$ and normalized to the housekeeping gene RSP18. One-way ANOVA, Dunnet's multiple comparisons test. ns, not significant. Each dot represents an independent experiment.
(C), (D) Activation of JKT cells expressing **(C)** the MR1-reactive Vγ9Vδ2 TCRs (D9A10, D1C55, G2B9) and (**D**) a MAIT TCR (SMC3) challenged with A375 β2m⁻ MR1 cells treated or not with Zol, 5-OP-RU, Ac-6-FP or vehicle. Bar plots of RLU mean ± SD of three independent experiments. Each dot represents one experiment. One-way ANOVA, Dunnet's multiple comparisons test. $^{**}p < 0.01$; $^{***}p < 0.001$; $^{****}p < 0.0001$.
(E) Flow cytometry plots of staining with anti-CD3 mAbs (x-axis) and MR1 tetramers (y-axis) on the indicated TCR Vγ9Vδ2 cell clones and TCR-transduced JKT cells. MR1 tetramers were loaded with different Ags (5-OP-RU, Ac-6-FP, M_1Ado, M_1dA, M_1Gua).

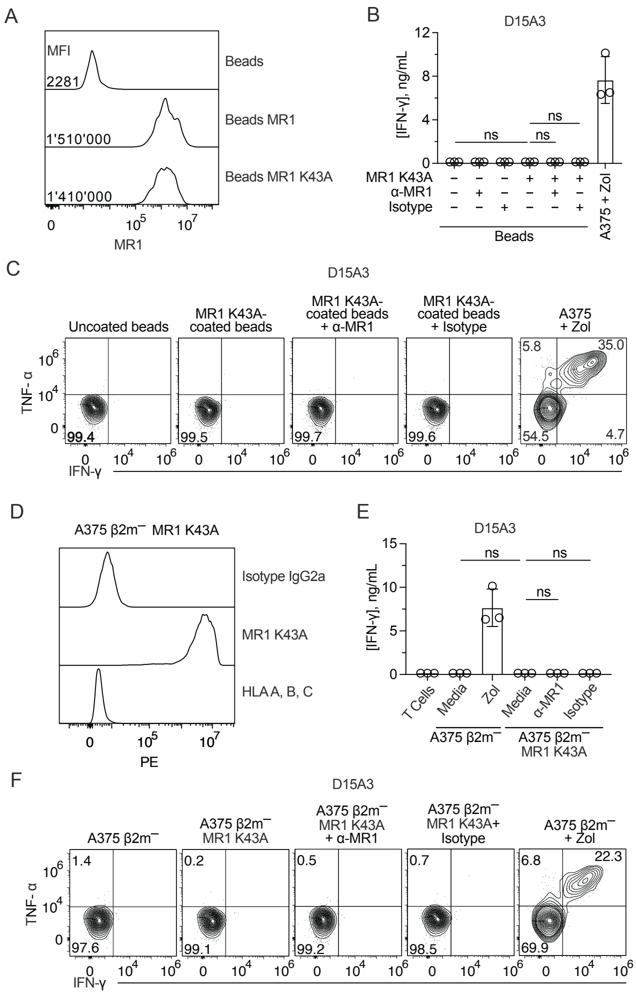

Supplementary Figure 7.

Mutant MR1 K43A does not stimulate the D15A3 clone.

(A) MR1 levels on the surface of uncoated beads (Beads), MR1- and MR1 K43A-coated beads. The median fluorescence intensity (MFI) is indicated on the left of each histogram. Data is representative of four independent experiments performed with different batches of MR1 molecules.

(B) Activation of D15A3 clone using beads coated or not with soluble MR1 K43A, in the presence or absence of anti-MR1 or isotype-matched mAbs. As a positive control, the clone was challenged with A375 cells treated with Zol (10μM). Data is representative of three independent experiments. Bar plots of IFN-γ release mean ±

SD of triplicate independent cultures. One-way ANOVA, Dunnett's multiple comparisons test. ns, not significant.

(C) Intracellular staining of IFN-γ and TNF-α in D15A3 clone after stimulation using beads coated or not with soluble MR1 K43A, in the presence or absence of anti-MR1 or isotype-matched mAbs. As a positive control, the clone was challenged with A375 cells treated with Zol (10μM). Data is representative of three independent experiments.

(D) MR1 and HLA A, B, C expression on A375 β2m⁻ MR1 K43A cells by flow cytometry.

(E) Activation of the D15A3 clone challenged with A375 β2m⁻ cells and A375 β2m⁻ MR1 K43A cells, in the presence or absence of anti-MR1 or isotype-matched mAbs. As a positive control, the clone was challenged with A375 cells treated with Zol (10μM). Data is representative of three independent experiments. Bar plots of IFN-γ release mean ± SD of triplicate independent cultures. One-way ANOVA, Dunnett's multiple comparisons test. ns, not significant.

(F) Intracellular staining of IFN-γ and TNF-α in D15A3 clone challenged with A375 β2m⁻ cells and A375 β2m⁻ MR1 K43A cells, in the presence or absence of anti-MR1 or isotype-matched mAbs. As a positive control, the clone was challenged with A375 cells treated with Zol (10μM). Numbers indicate percentages of cells in the quadrants. Data is representative of three independent experiments.

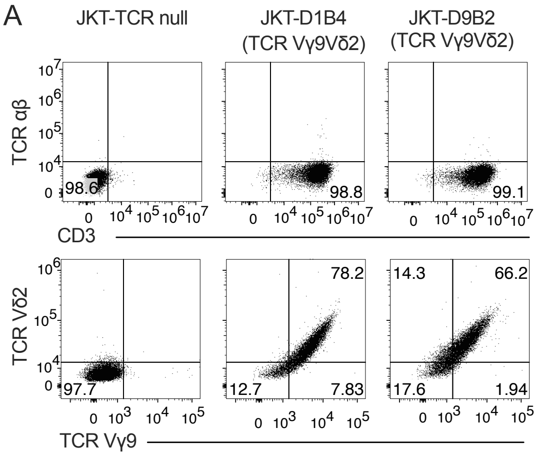

Supplementary Figure 8.

Generation of JKT cells expressing the same Vγ9 chain and different Vδ2 chain.

(A) Expression of two TCRs Vγ9Vδ2 (D1B4 and D9B2) on JKT cells. Flow cytometry plots of TCR αβ and CD3 (top plots) and Vδ2 and Vγ9 (bottom plots). Numbers indicate percentages of cells in the quadrants.

Supplementary Tables

Supplementary Table 1. Antibodies used in flow cytometry studies.

Conjugated mAbs specific for human surface markers				
**Target**	**Clone**	**Fluorochrome**	**Provider**	**Catalog Number**
MR1	26.5	PE	Biolegend	361105
MR1	26.5	APC	Biolegend	361108
HLA, A,B,C	W6/32	PE	Biolegend	311406
HLA, A,B,C	W6/32	APC	Biolegend	311410
Isotype IgG2a	MOPC-173	PE	Biolegend	981910
Isotype IgG2a	MOPC-173	APC	Biolegend	981906
CD3ε	SK7	PerCP-Cy5.5	Biolegend	344807
CD3ε	UCHT1	BUV805	BD Biosciences	612896
CD3ε	UCHT1	APC	Biolegend	300411
CD3ε	UCHT1	AF488	Biolegend	300454
TCR αβ	IP26	PE	Biolegend	984702
TCR γδ	B1	PE-Dazzle 594	Biolegend	331225
TCR Vγ9	B3	PerCP-Cy5.5	Biolegend	331321
TCR Vδ1	TS-1	FITC	Thermo Scientific	TCR2055
TCR Vδ2	B6	BV711	Biolegend	331412
TCR Vδ2	B6	FITC	Biolegend	331405
TCR Vδ2	B6	APC	Biolegend	331418
TCR Vδ3	D3P11.5B	Purified	Beckman Coulter	Out of catalog
CD4	OKT4	AF700	Biolegend	317425
CD8	RPA-T8	BUV496	BD Biosciences	612442
CD137	4B4-1	PE-Cy7	Biolegend	309818
CD137	4B4-1	APC	Biolegend	309810
CD137	4B4-1	APC-Cy7	Biolegend	309830
CD69	FN50	BV605	Biolegend	310938
CD69	FN50	PE	Biolegend	310906
CD69	FN50	PE-Cy7	Biolegend	310912
CD25	BC96	APC	Biolegend	302610
CD25	BC96	PE	Biolegend	302606
CD57	HNK-1	PerCP-Cy5.5	Biolegend	359622
CD28	CD28.2	AF647	Biolegend	302954

CD14	63D3	APC-Cy7	Biolegend	367108
CD19	H1B19	APC-Cy7	Biolegend	302218
CD26	M-A261	BV480	BD Biosciences	746696
KLRG1	2F1/KLRG1	APC	Biolegend	138412
CD95	DX2	BV650	Biolegend	305642
CD161	DX12	BUV737	BD Biosciences	748948
CD56	5.1H11	BV785	Biolegend	362550
NKp80	5D12	PE	Biolegend	346706
PD-1	EH12.2H7	BV421	Biolegend	329920
TIGIT	741182	BUV395	BD Biosciences	741182
CCR7	3D12	BUV563	BD Biosciences	741317
CD45RA	5H9	BUV661	BD Biosciences	741654
Conjugated mAbs specific for human intracellular cytokines				
**Target**	**Clone**	**Fluorochrome**	**Provider**	**Catalog Number**
IL-2	5344.111	BV650	BD Biosciences	563467
IL-4	MP4-25D2	BV510	Biolegend	500836
IL-10	JES3-9D7	PE-Cy7	Biolegend	501420
IL-13	JES10-5A2	PE	Biolegend	501903
IL-17A	BL168	AF488	Biolegend	512308
GM-CSF	BVD2-21C11	Pacific Blue	Biolegend	502314
IFN-γ	4S.B3	APC	Biolegend	502512
IFN-γ	4S.B3	BUV737	BD Biosciences	612845
TNF-α	MAb11	PE-Cy7	Biolegend	502930
TNF-α	MAb11	BV785	Biolegend	502948
Conjugated mAbs specific for mouse surface markers				
**Target**	**Clone**	**Fluorochrome**	**Provider**	**Catalog Number**
CD3ε	145-2C11	FITC	Biolegend	100306
CD137	17B5	PE	Biolegend	106105
CD25	PC61	APC	Biolegend	102012
CD69	H1.2F3	PE-Cy7	Biolegend	104512
Conjugated MR1/CD1D tetramers for TCR staining				
**Presenting molecule**	**Antigen**	**Fluorochrome**	**Provider**	**Catalog Number**
MR1	5-OP-RU	PE	NIH Tetramer Core Facility	33021

Supplementary Table 2. Immunoglobulin levels.

		Year of clinical evaluation		
**Immunoglobulins**	**Reference**	**2015**	**2019**	**2024**
IgG (g/l)	7.0 – 16.0	13.2	12.5	12
IgA (g/l)	0.70 – 4.00	2.28	2.30	1.88
IgM (g/l)	0.40 – 2.30	0.67	0.46	0.52
IgD (IU/ml)	<100	<23	---	---
IgE (IU/ml)	<100	30	---	30

Supplementary Table 3. Patient's hemogram.

		Year of clinical evaluation		
	**Reference**	**2018**	**2019**	**2024**
Leukocytes ($x10^9/l$)	3.5 – 10	5.82	10.66	7.14
Erythrocytes ($x10^{12}/l$)	4.5 – 6.3	5.33	5.30	5.21
Hemoglobulin (g/l)	140 – 180	168	169	166
Hematocrit (l/l)	0.38 – 0.52	0.48	0.48	0.47
MCV (fl)	79 – 95	91	91	90
MCH (pg)	27 – 33.2	31.5	31.8	31.8
MCHC (g/l)	320 – 360	347	350	354
EVB (%)	11.5 – 14.5	12.7	12.3	12.8
Hypochrome Ec (%)	0 – 5	0.1	0.4	0.1
Thrombocytes ($x10^9/l$)	150 – 450	224	297	181
MTV (fl)	6 – 10	7	6	7
TVB (%)	39 – 68	47.7	46.5	41.0
Reticulocytes (%)	10 – 27	16	--	--
Reticulocytes absolute ($x10^9/l$)	40 – 140	83	--	--
Mature reticulocytes (%)	80 – 96	89	--	--
Immature reticulocytes (%)	4 – 17	11	--	--
Reticulocytes CHR (pg)	27 – 33	33.6	--	--
Neutrophils (Seg+Stab) (%)	40 – 74	50.6	55.7	53.5
Neutrophils absolute (Seg+Stab) ($x10^9/l$)	1.3 – 6.7	2.94	5.94	3.82
Lymphocytes (%)	19 – 48	37.9	35.1	35.2
Lymphocytes absolute($x10^9/l$)	0.9 – 3.3	2.21	3.74	2.51
Monocytes (%)	3.4 – 9	7.0	5.3	6.7
Monocytes absolute ($x10^9/l$)	0.12 – 0.62	0.41	0.57	0.48
Eosinophils (%)	0 – 7	2.3	2.0	2.0
Eosinophils absolute ($x10^9/l$)	0 – 0.3	0.14	0.21	0.14
Basophils (%)	0 – 1.5	0.5	0.6	0.3
Basophils absolute ($x10^9/l$)	0 – 0.09	0.03	0.07	0.02
LUC (%)	0 – 4	1.6	1.3	2.2

LUC absolute ($x10^9$/l)	0 – 0.31	0.09	0.14	0.16
PMN (%)		55.5	56.6	59.5

Supplementary Table 4. Blood lymphocyte subpopulation.

		Year of clinical evaluation		
**Lymphocytes**	**Reference**	**2018**	**2019**	**2024**
T cells (cells/µl)	742 – 2750	2148	3132	2274
T cells (% of lymphocytes)	55 – 86	80	77	80
CD4+ T cells (cells/µl)	404 – 1612	631	1021	590
CD4+ T cells (% of lymphocytes)	33 – 58	24	24	21
CD8+ T cells (cells/µl)	220 – 1129	1262	851	1085
CD8+ T cells (% of lymphocytes)	13 – 39	47	20	38
B cells (cells/µl)	80 – 616	218	426	198
B cells (% of lymphocytes)	5 – 22	8	11	7
NK cells (cells/µl)	84 – 724	276	443	331
NK cells (% of lymphocytes)	5 – 26	10	11	12
